# Comparing perceptions of users on digital authentication through one-time passcode, fingerprint, voice recognition, PIN code, finger swipe, and authentication of choice: A cross-sectional survey

**DOI:** 10.1371/journal.pone.0344162

**Published:** 2026-04-01

**Authors:** Alexis Bennett, Elochukwu Ukwandu

**Affiliations:** 1 Department of Applied Psychology, Cardiff School of Sports and Health Sciences, Cardiff Metropolitan University, Cardiff, Wales, United Kingdom; 2 Department of Applied Computing, Cardiff School of Technologies, Cardiff Metropolitan University, Cardiff, Wales, United Kingdom; University of North Texas, BANGLADESH

## Abstract

The use of passwords for end-user authentication has been fraught with issues for decades, making passwordless authentication (PLA) systems a needed alternative for password-based authentication (PBA). PLA systems involve any methods that help identify a user without the use of a password – methods that are often rolled out with trade-offs in security, privacy, and convenience as means of innovation. Meanwhile, successful implementation of these systems is dependent on their acceptance, and data on the views of the users have not yet fully covered the wide range of approaches and contexts. The current study explored the perceptions of the users ranging from occasional information technology (IT) users to professional IT developers through a cross-sectional survey, assessing their views across different authentication methods (one-time passcode (OTP), fingerprint (FP), voice recognition (VR), personal identification number (PIN), finger swipe (FS), and authentication of choice (AoC)) in different contexts (low-risk account login and payment confirmation). One hundred seventy participants, aged 18–65 years and representing five different levels of IT experience contributed to the survey. The results shed light on the perceptions, concerns, and preferences of the users on PLA and PBA through quantitative and qualitative data, suggesting that in both use scenarios, OTP, fingerprint and PIN formed a cluster of favourites, followed by AoC; common reasons were convenience, usability, reliability and accuracy. However, knowledge gaps and misconceptions were present, highlighting the importance of carefully designed, adjusted, and targeted user information. Future research could extend the investigation to larger samples and more narrow-focused and refined survey items to further explore, for example, the finer differences between OTP, FP, and PIN, and on the other hand, VR and FS. The current findings are expected to benefit the industry involved in end-user authentication by providing empirical evidence on the views of corporate and end users on these authentication systems; by influencing the choice of use cases and methods deployed by software and system developers; and by enhancing the knowledge of professionals and industry experts specialising in user experience design and identity and access management.

## Introduction

### Surge in passwordless authentication

Passwordless authentication (PLA) involves methods that help to identify a user in computer-based systems without the use of an actual password. Particularly, PLA research has focused on the application of attributes, individual knowledge and possessions, public-key cryptosystems (cryptographic key pairs) [1], inherent factors (biometrics) [2], and Email Magic links (using a link sent to email for logging in) [3], as means of unique user identification. The development and roll-out of PLA systems have been seen as the future of user access management [4], and recent developments have increased their prominence. For example, Ankit and Vineet [5] suggest that the emergence of the COVID-19 pandemic led to an increase in automation that resulted to a change in the dynamics of cyber threats [6], and as a result of this, the use of PLA approaches, such as biometrics, is expected to surge. Due to the increasing needs of authentication within and between technological applications, this surge is expected to be further fuelled by the growth of the Internet of Things (IoT) technologies (with systems of networked devices requiring applicable and safe authentication) and machine learning [2] (also coupled to IoT) and artificial intelligence [6], along with the increase in the use of consumer electronic devices and enhancement of customer experience. As a further viewpoint, Lallie et al. [6] suggest that increasing awareness of passwordless authentication in the banking industry will enhance the adoption of these approaches.

### Advantages of passwordless authentication

The shift to a passwordless approach is comprehensible considering the frequency of issues related to password-based authentication (PBA). About 80% of malicious hacking incidents have been caused by stolen and reused login information resulting in about 555 million stolen passwords on the dark web as at 2021 [7]. Meanwhile, advantages of PLA include ease of use and uniqueness [8], protection against malicious attacks, enhanced user experience and improved security [9]: researchers such as Al Kabir & Elmedany [10], Alqubaisi et al. [11], Gordin et al. [12], and Haddad et al. [13] suggest that PLA use cases are not susceptible to the inherent challenges, vulnerabilities, and limitations that come with the use of PBA systems. Although PLA has its own challenges, such as cost of deployment; limited account recovery options; risk of biometric and possession-based authentication failure, biometric data leaks, and loss of authenticator; and vulnerability to attacks when using physical and software-based tokens [8,10,14,15], it is considered a promising alternative to passwords [16].

### Importance of user views and the role of trade-offs in PLA implementation

Despite the points discussed, the use of passwords persists [1] while the uptake of PLA has been slow and low [1,17], driving the need for better understanding of user views. For example, Lyastani et al. [16] and Owens et al. [18] emphasise the role of integrating the views of end-users in the development of digital device authentication in enhancing their uptake and adoption. Similarly, Oogami et al. [19], Bhagavatulas et al. [20], Cherapau et al. [21], and Wolf et al. [22] highlight the importance of incorporating end-users’ views when tackling usability issues in authentication. A key aspect in this discussion is the need for trade-offs, such as trading security for convenience or usability [5,22–24] – essentially aiming to find the best balance between factors such as availability, speed, ease of use, reliability, or low vulnerability. For example, one-time passcode (OTP) [25] provides moderate security and a good level of convenience but may be slow or fail due to its network dependency and is not usually recommended as the only factor for end-user authentication for high-value assets. Meanwhile, fingerprint (FP) is convenient, fast, and can offer robust security through biometric verification, while it is potentially vulnerable to presentation attacks [26]. Voice-based authentication [27], in turn, is easily accessible but environmentally sensitive and susceptible to replay/deepfake risk and therefore better used as another layer of security or within multi-modal systems. PIN code systems are fast and easy when it comes to end-user authentication; however, they have the lowest entropy, are easily susceptible, and are vulnerable to observation-based attack and smudge [28]. Meanwhile, PIN codes are more secure when used as hard tokens rather than soft tokens, though application areas differ [29].

Overall, balancing these aspects has become a factor researchers and developers highly value, but achieving this requires keeping the perceptions of the end-users at the centre of the design, development, implementation, and roll-out.

### User views of digital authentication

While the views of the users are important in the development of end-user authentication methods, profound coverage of users’ views on the wide range of possible methods including Authentication of Choice (AoC) – the user’s choice at the point of authentication – has been scarce [30]. Research on AoC is still limited but the concept is here used in the sense applied by Arinde et al. [30], referring to giving users the freedom to select one or more authentication methods of their own choice from a set of provided options. This approach is of particular interest as it has been indicated as potentially advantageous regarding the balance between security and usability [30] Overall, at the point of writing, the existing data of user views regarding individual PLA methods suggests, in general, a level of readiness, with accompanying concerns. For example, in Oogami et al.’s [19] usability study on phone fingerprint in website-based registration and authentication in Japan, many participants expressed a desire to use fingerprint authentication and, after experiencing its benefits, an intention of regular use, while the setup process was perceived challenging and the interface confusing [19]. Visa [31], cited in [2]) supported growing interest and openness to biometric authentication, among the Swiss population, as about 85% preferred fingerprint authentication as the most secure means of credit card payments. Lyastani et al. [16] explored users’ perceptions, acceptance and concerns around a FIDO2 solution, Yubico security key. A general preference was identified towards PLA systems involving security keys in terms of usability and acceptability, while these views were accompanied by concerns ranging from authenticator loss to malicious actors, issues with account recovery, and limited application platforms [16]. Meanwhile, Kruzikova et al. [32] explored mobile banking and the users’ perceptions of the usability and security of four methods – fingerprint, PIN, token, and card reader – finding all positively evaluated for both usability and security, while fingerprint verification was rated highest for both aspects. Cho et al. [33], in turn, explored multiple knowledge-based and biometric-based authentication choices (fingerprint, PIN, swipe pattern, face recognition, iris scanner, password, voice recognition) for unlocking and rebooting mobile phones. Fingerprint had a monopolistic dominance amongst biometric-based methods for phone unlocking; meanwhile, providing both knowledge-based and biometric-based authentication categories increased convenience, as a combination of these was frequently used [33]. Among the options, both PIN and swipe patterns were fairly evenly used [33]. Arinde et al. [30] involved, as options for AoC, five authentication methods (password, PIN, OTP, fingerprint, and face recognition), and found PIN to be clearly the most frequently selected method, followed – by distance – by fingerprint, after which OTP and face recognition competed for the next place depending on whether one- or two-factor authentication was used. Providing a different example, Renz et al. [34] focused on voice authentication and explored spoken PIN, biometric recognition, and confirmation via an app using a button or voice, compared with the use of a card reader. They found a general preference for additional confirmation of voice-based instructions and for the use of a card reader for high security contexts, while voice recognition as such was considered low in reliability [34]. Meanwhile, the inconvenience of card reader authentication was recognised [34].

### Survey: Views of digital authentication across methods and contexts

Preliminarily, PLA approaches seem generally favourably viewed, but more information on the related concerns are needed to increase acceptance and uptake of the different approaches. In addition, further data is needed to better cover and compare user views on a range of digital authentication solutions in different specific contexts of authentication with varying security implications – for example, online store account login situations versus online banking or payment events. To provide insight into the perceptions of users on a range of authentication approaches in different contexts and to support ongoing and future research on and development of digital authentication systems, the current survey explored the views and perceptions of end-users in a cross-sectional online survey. Investigated aspects included the choice and ranking of authentication methods for different purposes, the rationale behind the choices, and the concerns and preferences with respect to the methods, including also AoC and addressing the lack of data noted by Arinde et al. [30]. To consider several authentication types, the compared methods involved approaches based on known details (knowledge factors), accessible details (possession factors), and biometrics: One-time passcode (OTP), fingerprint (FP), voice recognition (VR), personal identification number (PIN), and finger swipe (FS). Meanwhile, with authentication of choice (AoC) any of the methods could be selected. The specific contexts were a lower risk setting of casual account login (for example, logging into an online shop account) and a higher risk context of payment confirmation. Both scaled responses and free-text expressions were collected and analysed.

### Contributions

To the best of the authors’ knowledge, this study is the first published account combining end-users’ familiarity, frequency of use, preferences, ranking, and perceptions of benefits and concerns regarding one-time passcode, fingerprint, voice recognition, personal identification number code, finger swipe and authentication of choice in the contexts of end-user account login and payment confirmation. The authors expect that the findings from the current study will provide insight on the end-users’ attitudes towards, and experiences of, different end-user authentication approaches, which is expected to be useful in the development and roll-out of responsibly innovative authentication systems in this domain. Given that both individuals and corporate entities that rely on IT tools for their services or engagements undergo end-user authentication processes before gaining access to their digital assets, the field of application is wide and the results can benefit both the public and the corporate environments. Taking the current findings into account in the development of the methods has the prospect of leading to a wider adoption and utilisation of new authentication systems, especially PLA approaches, in digital systems.

## Methods

The reporting of this survey was guided by the Consensus-Based Checklist for Reporting of Survey Studies (Sharma et al., [35]). This was a cross-sectional online survey conducted in Qualtrics from 15/03/2023 to 30/06/2023.

### Survey development and structure

The questionnaire was developed in collaboration between the research team and the industry partner MIRACL, guided by Krosnick and Presser [36], Vannette [37], Venkatesh et al. [38], and existing literature on exploring user views of authentication, such as Pedersen [29]; Weir et al. [23]; and Wolf et al. [22]. The questionnaire was assessed in two rounds of pilot testing; the pilot group involved end-users of authentication with little or no experience in computing as well as researchers from the fields of computing, psychology, and authentication development. Based on the feedback, the questionnaire was adjusted for ease of use, structure, content, and clarity.

The questionnaire first presented the authentication methods – (i) one-time passcode, (ii) fingerprint, (iii) voice recognition, (iv) personal identification number, and (v) finger swipe – one at a time and asked a set of questions about each method before proceeding to the next. The presentation of each method included a short description of a typical use scenario and any prior setup requirements for the user. A representative image was used to help recognise and remember the method later in the survey (see S3 Appendix). The first method (i) was selected so that it was relatively common and estimated to be effortless to recognise and evaluate. This aimed to help participants engage with the survey and evaluate other, expectedly less familiar, methods later. Methods with similarities – OTP (i)/ PIN (iv), and fingerprint (ii)/ finger swipe (v) – were separated in presentation order to avoid confusion. Potential effects of the order of presentation in this section were considered in the analysis; meanwhile, in the remaining questions the methods were presented in random order. For each method, the participants were asked how familiar they were with the method, how frequently they used it, and how happy they would be to accept the method for the purposes of (a) online shop account login and (b) payment confirmation, and why they would be happy/unhappy (as applicable) to do this (Table 1/ *Assessment of individual methods*). All evaluation questions included the option of a free text answer, and the evaluation of each method ended with a final open question of whether the participants had anything to add about the method. To facilitate the method evaluation and the completion of the survey, the rationale for the acceptance/rejection was asked as a multiple choice question involving a selection of predefined options designed with the pilot group and guided by the literature (Table 1) and a free text option. To further facilitate the completion of the survey, the aspects *reliability* and *security* were combined as a single option at this point of the survey, while in the later stages and in the free text comments these were analysed separately.

**Table 1:** Questionnaire instruments for assessing digital device authentication methods.

Assessment of individual methods			
Instrument	Content	Scale	Options
Familiarity	Familiarity with the method	5-point Likert	Not at all... Extremely
Frequency	Frequency of use of the method	5-point Likert + uncertainty option	Never … 5–7 days a week; Not sure
Acceptance (login) *	Willingness to use for low-risk customer account login	7-point Likert	Very unhappy... Very happy
Acceptance (payment) *	Willingness to use for payment confirmation	7-point Likert	Very unhappy... Very happy
Rationale (login)	Rationale for willingness to use for low-risk customer account login	Multiple choice	**
Rationale (payment)	Rationale for willingness to use for payment confirmation	Multiple choice	**
Benefits and concerns	Any positive/ negative aspects of the method, if non-acceptance/ acceptance	Multiple choice	**
Comments	Further comments on the method	Open question	Free text
**Ranking and preference of methods**			
**Instrument**	**Content**	**Scale**	**Options**
Ease of use	Ranking of methods in order of preference regarding ease of use	Rank order	Text 1–5***
Security	Ranking of methods in order of preference regarding security	Rank order	Text 1–5***
Preference (login)	Preferred methods for low-risk customer account login	Multiple choice	OTP, FP, VR, PIN, FS, AoC
Preference (payment)	Preferred methods for payment confirmation	Multiple choice	OTP, FP, VR, PIN, FS, AoC

* Response required.

** As applicable: complexity/ ease of use, convenience, device dependency, familiarity, reliability or security, requirements, speed; Uncertainty option; Other suggestions (free text).

*** Ranking: Highest regard = 1, lowest = 5; Shared rankings and non-rankings accepted.

The next sections asked the participants to rank the methods according to their preferences in terms of two core concepts in authentication [23] – ease of use (usability) and security – and to indicate their preferred methods regarding (a) logging into an online shop and (b) payment confirmation, including AoC in the range of possible options (Table 1/ *Ranking and preference of methods*). The final section of the survey collected their demographic data (gender and age), level of IT use (see *Expertise on IT use* below), and any further free-text comments.

The survey instruments involved 5- and 7- point Likert scales and multiple choice, ranking, and free-text questions (Table 1). The order of the options in the Likert scales was arranged from unfavourable to favourable to avoid response order bias [36]. To increase accessibility, separate multiple-choice questions and textbox ranking were used instead of matrix questions and visual ranking.

### Assessment of authentication methods

The authentication methods were evaluated individually, ranked by ease of use and security, and assessed regarding preference for low-risk login and for payment confirmation, including the option to choose at the point of authentication; the instruments for assessment are presented in Table 1 and the full wording of the questions is presented in the S1 Appendix.

### Expertise on IT use

The responses were observed against the respondents’ levels of expertise on IT use. This approach was guided by the United Theory of Acceptance and Use of Technology (UTAUT [38]), which models the influence of performance expectancy, effort expectancy, social influence, and facilitating conditions on user behaviour and choice, and research such as Wolf et al. [22] that demonstrates differences in the levels of acceptance of passwordless authentication between users with different levels of technological expertise.

The development of the scale of IT use was guided by the Skills Framework for the Information Age (SFIA [39]), the National standards for essential digital skills [40], and the national statistics of internet users in the United Kingdom [41]. The scale was designed in collaboration between the research team and the industry partner MIRACLE and developed based on the feedback in the pilot tests, adjusting for clarity and ease of use. The scale covered five levels of users in the target sample, from occasional IT use (Level 1) to a professional IT developer (Level 5) (see S2 Appendix for the full wording).

### Population and recruitment

The target population consisted of end-users of 18 years of age or older, with IT use experience as described above, suggesting at least some familiarity with digital authentication based on at least occasional use of IT and internet, and with no upper limit on IT experience. Recruitment was conducted via voluntary response sampling and snowball sampling through adverts on a university online discussion platform for all staff and undergraduate and postgraduate students (27.816 members, with approximately 20% actively reading the site (estimate at October 2024), in the full range of targeted IT expertise), and convenience and snowball sampling through the researchers’ and the industry partner MIRACL’s networks (the latter reaching approximately 3000 IT professionals and their networks through their own advertising and an unknown number through their related associations). While the exact size of the target population was unknown, the targets for confidence level, standard of deviation, and margin of error were set at minimum 90%; 0.5; and 8% or lower, respectively, following commonly acceptable ranges, leading to a target sample size of minimum 106 completed responses. Once this level of responses or higher had been reached, the data could be analysed. If the target was not reached in the target period March – June 2023, the data collection period could be extended (to a maximum of one year as defined in the ethical approval of the study).

### Ethical considerations and procedure

The study was approved by the Ethics Committee of [removed for review anonymity]. The survey was entirely anonymous as no personal identifiers were collected and any potential identifiable information in the free-text responses were anonymised. In addition, the participants had the opportunity to withdraw their responses within a seven-day withdrawal period. For this purpose, at the beginning of the survey, the participants created an anonymous withdrawal code by giving a sequence of four digits (unidentifiable and unguessable) of their own choice. By contacting the researchers (using an anonymous email account if they wished) and giving this code, they could remove their responses from the data set.

Participants started the survey by following an electronic link, after which they were presented with the participant information of the study and asked to tick the check boxes of an electronic consent form. This confirmed their informed consent to participate and was required before they could proceed to the study. Participants were then asked to create the withdrawal code as described above; presented with the authentication methods; and asked the evaluation and ranking questions, demographic data, and their level of IT use. They were offered the opportunity to freely comment on anything that was on their mind before submitting the survey. The duration of the survey was approximately 10 minutes.

### Analysis

Analysis of the data was conducted in two stages:

**Stage 1: Method evaluations**
  - **Assessments**: Participants’ assessment of the individual methods ((i) one-time passcode, (ii) fingerprint, (iii) voice recognition, (iv) pin code, (v) finger swipe) were observed, describing familiarity, frequency of use, acceptance for login purposes, acceptance for payment purposes, and rationale for the views, for each method. The Likert scale responses were coded into levels ranging from 1 to 5, 6, or 7 depending on the aspect observed, with higher numbers indicating higher regard for the method. The coding should be considered with the notion that Likert scales are ordinal and discrete and the distances between the levels are not quantifiable as absolute values; meanwhile, median levels and nominal mean values can be used for insight on where on the scale most answers lie, and analysis considering the order of the levels using nonparametric statistical tests is applicable. Free-text responses for each aspect were collated and assigned into emerging preliminary codes, which were grouped into emerging main themes [42]. The themes were described, and representative individual comments were extracted as examples.
  - **Rankings**: The participants’ ranking of the methods for account login and payment confirmation situations was explored. The rankings were analysed as given; therefore a low value (e.g., 1 = first) indicates high regard and a high value (e.g., 5 = last) indicates low regard (instruments *Ease of Use* and *Security*).
  - **Preferences:** The participants’ preferences of the individual methods and AoC were observed based on the methods selected by the participants for (a) account login and (b) payment confirmation (instruments *Preference (login)* and *Preference (payment)*). Selection was coded as 1 and non-selection as 0. The ratios of the participants that selected each method were observed, and for comparison purposes, the results were also converted onto scale 1–5.

**Statistical differences:** For each aspect above, due to ordinal data and within-subjects design, statistical differences between the methods were explored using, as a nonparametric alternative to the one-way repeated-measures ANOVA, the Friedman test, which is also applicable in dichotomous cases (e.g., selection/non-selection) where it essentially becomes Cochran’s Q test, an extension of Mc Nemar’s test. Due to asymmetrical distribution of differences between the paired observations, the paired-samples sign test was used for post-hoc analyses – similarly to Friedman, this test is applicable also in binary cases, where it becomes the exact Mc Nemar’s test. In case of very few data points when analysing binary data, binomial distribution was used instead of the sign test. To avoid Type I error (false positive) due to multiple comparisons, Bonferroni adjustment was used for significance level: significance level was set at p = .005 in case of ten comparison pairs (five methods) and p = .0033 in case of 15 comparison pairs (five methods and AoC).

**Stage 2: Associations**

Due to ordinal data, the relationships between the factors in the data were tested using Spearman rank-order correlation coefficient, observing the consistency of the answers and any links between the participants’ choices and the characteristics in the data. For the ranking data, the order of regard was opposite to the other questions (1 = first (highest regard), 5 = last (lowest regard)), while for other evaluation data, higher value indicated higher regard. For effect size estimates, Cohen’s thresholds of 0.1 (small effect size/ weak correlation)/ 0.3 (medium effect size/ moderate correlation)/ 0.5 large effect size/ strong correlation were applied to Spearman’s rho. (See Table 1 for the instrument descriptions.)

Consistency of answers was investigated between

- individual method evaluations (*Acceptance (login)* and *Acceptance (payment)*) and
- method preferences (*Preference (login)* and *Preference (payment)*).

Connection between *Familiarity* and *Frequency* was also tested.

Participants’ choices were observed through the questions of

- acceptance of methods (*Acceptance (login)* and *Acceptance (payment)*) and
- preferred methods (*Preference (login)* and *Preference (payment)*).

The factors investigated as potentially linked with participants’ choices included

- context (low or high risk (login or payment)),
- perceived *Security* and *Ease of use,*
- experience (*Familiarity* and *Frequency*), and
- participant characteristics (expertise on IT use; gender; age).

Associations between the last three categories (perceived *Security*/ *Ease of use*, experience, and participant characteristics) were also tested.

## Results

### Respondent characteristics

Of the 204 participants who followed the survey link, 170 participants (83.3%) gave their consent and continued with the questions. Of these participants, 141 (82.9%) completed the full survey, leading to a general margin of error of 8.42% at 95% confidence level. For the remaining 29 participants, the average progress in the survey was 28.8%. The age range was 18 to 65 years, 36.9% women, 60.3% men (N = 141). Participants’ level of IT use varied in the full range of expertise (1 to 5), while most participants reported levels 2–5 with the mean slightly above average, at 3.3 (see Table 2). The relatively small representation (5%) at the low end of IT experience is in line with the estimates of non-recent internet users in the United Kingdom (1–7% in the range 16–64 years) by the Office for National Statistics [41]. Seasonal variation during data collection was limited (late spring – early summer) and no public incidents or other external phenomena that might have influenced the results were observed.

**Table 2:** Participants’ level of use of information technology.

Level of IT use	Participants	Percentage
Level 1	7	5.0%
Level 2	31	22.0%
Level 3	39	27.7%
Level 4	34	24.1%
Level 5	28	19.9%
Prefer not to say	2	1.4%
	141	100.0%

### Stage 1: Method evaluations

#### Assessment of authentication methods.

The participants’ evaluations for familiarity, frequency of use, and acceptance for account login and payment confirmation across the methods are presented in Table 3. Participants’ rationales for their evaluations are presented in Table 4. Participants’ free-text answers are explored below together with the numeric data, for each method.

**Table 3:** Reported familiarity, frequency of use, and acceptance for login and payment purposes, across authentication methods.

Aspect	Scale level/ Measure	One-time passcode	Fingerprint	Voice recognition	PIN code	Finger swipe
**Familiarity**	Not familiar at all (1)	3.6% ± 2.8%	5.3% ± 3.6%	37.6% ± 7.8%	0% ± 0%	23.2% ± 6.9%
	Slightly familiar (2)	3.6% ± 2.8%	7.3% ± 4.1%	26.2% ± 7.1%	2.8% ± 2.7%	19.7% ± 6.5%
	Moderately familiar (3)	3.6% ± 2.8%	7.3% ± 4.1%	14.1% ± 5.6%	4.2% ± 3.3%	18.3% ± 6.4%
	Very familiar (4)	42.6% ± 7.5%	43.1% ± 7.9%	15.4% ± 5.8%	36.4% ± 7.9%	28.9% ± 7.5%
	Extremely familiar (5)	46.8% ± 7.5%	37.1% ± 7.7%	6.7% ± 4%	56.6% ± 8.1%	9.9% ± 4.9%
	Median (N total)	4	4	2	5	3
	Mean (N total)	4.3 ± 0.1	4.0 ± 0.2	2.3 ± 0.2	4.5 ± 0.1	2.8 ± 0.2
	SD (N total)	0.95	1.11	1.29	0.71	1.34
	N total	169	151	149	143	142
	Median (N compared)	4	4	2	5	3
	Mean (N compared)	4.3 ± 0.1^a,b^	4.0 ± 0.2^a^	2.3 ± 0.2	4.5 ± 0.1^b^	2.8 ± 0.2
	SD (N compared)	0.841	1.089	1.306	0.713	1.334
	N compared	141	141	141	141	141
**Frequency**	Never (1)	3.6% ± 2.8%	11.3% ± 5.1%	60.4% ± 7.9%	1.4% ± 2%	38.7% ± 8%
**of use**	Once or twice (2)	4.7% ± 3.2%	10.7% ± 4.9%	22.8% ± 6.7%	0.7% ± 1.4%	12% ± 5.3%
	Several times (3)	36.7% ± 7.3%	24% ± 6.8%	8.7% ± 4.5%	23.4% ± 7%	23.2% ± 6.9%
	Frequently (4)	46.8% ± 7.5%	40.7% ± 7.9%	4% ± 3.2%	54.6% ± 8.2%	18.3% ± 6.4%
	5 to 7 days a week (5)	4.7% ± 3.2%	10% ± 4.8%	0.7% ± 1.3%	18.4% ± 6.4%	2.8% ± 2.7%
	Not sure	3.6% ± 2.8%	3.3% ± 2.9%	3.4% ± 2.9%	1.4% ± 2%	4.9% ± 3.6%
	Median (N total)	4	4	1	4	2
	Mean (N total)	3.5 ± 0.1	3.3 ± 0.2	1.6 ± 0.1	3.9 ± 0.1	2.3 ± 0.2
	SD (N total)	0.82	1.16	0.87	0.76	1.27
	N total	169	150	149	141	142
	Median (N compared)	4	4	1	4	2
	Mean (N compared)	3.5 ± 0.1^a^	3.3 ± 0.2^a^	1.6 ± 0.2	3.9 ± 0.1	2.4 ± 0.2
	SD (N compared)	0.74	1.18	0.87	0.78	1.28
	N compared	124	124	124	124	124
**Acceptance**	Very unhappy (1)	2.4% ± 2.3%	2% ± 2.2%	16.1% ± 5.9%	2.1% ± 2.4%	14.8% ± 5.8%
**for**	Unhappy (2)	7.1% ± 3.9%	6% ± 3.8%	20.8% ± 6.5%	2.1% ± 2.4%	10.6% ± 5.1%
**login**	Somewhat unhappy (3)	10% ± 4.5%	2% ± 2.2%	14.1% ± 5.6%	4.2% ± 3.3%	13.4% ± 5.6%
	Neither happy nor unhappy (4)	14.1% ± 5.2%	11.3% ± 5%	26.9% ± 7.1%	18.9% ± 6.4%	30.3% ± 7.6%
	Somewhat happy (5)	21.2% ± 6.1%	26.5% ± 7%	9.4% ± 4.7%	22.4% ± 6.8%	12.7% ± 5.5%
	Happy (6)	28.2% ± 6.8%	25.2% ± 6.9%	10.7% ± 5%	35% ± 7.8%	14.8% ± 5.8%
	Very happy (7)	17.1% ± 5.7%	27.2% ± 7.1%	2% ± 2.3%	15.4% ± 5.9%	3.5% ± 3%
	Median (N total)	5	6	3	6	4
	Mean (N total)	5.0 ± 0.2	5.4 ± 0.2	3.3 ± 0.3	5.2 ± 0.2	3.7 ± 0.3
	SD (N total)	1.59	1.50	1.64	1.34	1.70
	N total	170	151	149	143	142
	Median (N compared)	5	6	3.5	6	4
	Mean (N compared)	5.0 ± 0.3^a^	5.4 ± 0.2^a^	3.4 ± 0.3^b^	5.3 ± 0.2^a^	3.7 ± 0.3^b^
	SD (N compared)	1.60	1.45	1.66	1.34	1.70
	N compared	142	142	142	142	142
**Acceptance**	Very unhappy (1)	2.4% ± 2.3%	2% ± 2.2%	22.2% ± 6.7%	1.4% ± 1.9%	16.2% ± 6.1%
**for**	Unhappy (2)	7.1% ± 3.9%	8% ± 4.3%	22.8% ± 6.7%	2.8% ± 2.7%	17.6% ± 6.3%
**payment**	Somewhat unhappy (3)	4.7% ± 3.2%	1.3% ± 1.8%	16.8% ± 6%	2.1% ± 2.4%	12.7% ± 5.5%
**confirmation**	Neither happy nor unhappy (4)	9.4% ± 4.4%	12.6% ± 5.3%	19.5% ± 6.4%	19.6% ± 6.5%	29.6% ± 7.5%
	Somewhat happy (5)	19.4% ± 5.9%	24.5% ± 6.9%	10.1% ± 4.8%	23.1% ± 6.9%	9.9% ± 4.9%
	Happy (6)	33.5% ± 7.1%	21.9% ± 6.6%	7.4% ± 4.2%	31.5% ± 7.6%	12.7% ± 5.5%
	Very happy (7)	23.5% ± 6.4%	29.8% ± 7.3%	1.3% ± 1.8%	19.6% ± 6.5%	1.4% ± 1.9%
	Median (N total)	6	6	3	6	4
	Mean (N total)	5.3 ± 0.2	5.3 ± 0.3	3 ± 0.3	5.3 ± 0.2	3.4 ± 0.3
	SD (N total)	1.58	1.59	1.61	1.33	1.65
	N total	170	151	149	143	142
	Median (N compared)	6	6	3	6	4
	Mean (N compared)	5.3 ± 0.2^a^	5.4 ± 0.3^a^	3.0 ± 0.3^b^	5.3 ± 0.2^a^	3.4 ± 0.3^b^
	SD (N compared)	1.52	1.52	1.61	1.33	1.65
	N compared	142	142	142	142	142

Margins of error at 95% confidence level. a, b: Methods sharing the same letter are statistically similar.

(N total): Data from all participants who evaluated the aspect, for the specific method.

(N compared): Data from participants who evaluated the same aspect for all five methods.

**Table 4:** Reported rationale for estimates, across authentication methods.

Aspect	Response option	One-time passcode	Fingerprint	Voice recognition	PIN code	Finger swipe
**Rationale:**	Ease of use	17.8%	19.1%	12.5%	19.1%	22.3%
**Account login,**	Convenience	19.1%	24.2%	25.0%	23.2%	25.5%
**acceptance**	Low device dependency	7.6%	2.7%	3.1%	4.8%	2.1%
	Familiarity	7.6%	9.1%	10.9%	16.2%	13.8%
	Reliability or security	28.4%	21.1%	23.4%	16.5%	11.7%
	Speed	15.1%	21.1%	20.3%	19.5%	22.3%
	Other	3.6%	1.7%	0.0%	0.7%	0.0%
	Not sure	0.9%	1.0%	4.7%	0.0%	2.1%
	Total of stated reasons	225	298	64	272	94
	N	112	124	32	106	43
**Rationale:**	Complexity	4.0%	17.7%	12.9%	0.0%	8.3%
**Account login,**	Inconvenience	16.0%	11.8%	17.9%	5.9%	6.2%
**non-acceptance**	Device dependency	26.0%	23.5%	6.4%	5.9%	14.4%
	Familiarity	4.0%	0.0%	6.4%	11.8%	7.2%
	Reliability or security	16.0%	29.4%	32.1%	41.2%	37.1%
	Setup requirements	2.0%	5.9%	6.4%	0.0%	9.3%
	Speed	16.0%	0.0%	6.4%	5.9%	6.2%
	Other	14.0%	11.8%	9.3%	29.4%	5.2%
	Not sure	2.0%	0.0%	2.1%	0.0%	6.2%
	Total of stated reasons	50	17	140	17	97
	N	39	16	86	17	60
**Rationale:**	Ease of use	16.3%	14.0%	15.5%	18.7%	16.2%
**Payment**	Convenience	17.9%	25.9%	25.9%	22.2%	27.0%
**confirmation,**	Low device dependency	6.7%	3.2%	8.6%	4.8%	4.1%
**acceptance**	Familiarity	10.8%	11.5%	8.6%	18.3%	12.2%
	Reliability or security	31.7%	24.5%	20.7%	17.3%	17.6%
	Speed	13.8%	18.5%	17.2%	17.7%	20.3%
	Other	1.7%	1.1%	0.0%	0.7%	0.0%
	Not sure	1.3%	1.4%	3.5%	0.4%	2.7%
	Total of stated reasons	240	286	58	289	74
	N	123	117	27	107	34
**Rationale:**	Complexity	3.3%	4.6%	11.6%	0.0%	6.3%
**Payment**	Inconvenience	13.3%	13.6%	20.2%	9.1%	9.0%
**confirmation,**	Device dependency	10.0%	18.2%	9.3%	0.0%	14.4%
**non-acceptance**	Familiarity	6.7%	0.0%	8.7%	0.0%	6.3%
	Reliability or security	30.0%	31.8%	28.3%	54.6%	38.7%
	Setup requirements	3.3%	9.1%	8.1%	9.1%	9.9%
	Speed	13.3%	0.0%	5.2%	0.0%	5.4%
	Other	16.7%	13.6%	5.8%	27.3%	3.6%
	Not sure	3.3%	9.1%	2.9%	0.0%	6.3%
	Total of stated reasons	30	22	173	11	111
	N	26	19	98	12	70
**Any positive**	Ease of use	16.0%	6.3%	6.9%	20.0%	16.7%
**aspects**	Convenience	12.0%	6.3%	8.1%	10.0%	10.7%
**when**	Low device dependency	16.0%	0.0%	4.6%	0.0%	4.8%
**non-acceptance**	Familiarity	20.0%	12.5%	4.6%	10.0%	8.3%
**in both contexts**	Reliability or security	16.0%	25.0%	2.3%	0.0%	3.6%
	Speed	4.0%	18.8%	8.1%	20.0%	16.7%
	Nothing positive	4.0%	12.5%	37.9%	10.0%	21.4%
	Other	8.0%	12.5%	4.6%	10.0%	1.2%
	Not sure	4.0%	6.3%	23.0%	20.0%	16.7%
	Total of stated reasons	25	16	87	10	84
	N	17	13	73	7	54
**Any negative**	Complexity	2.3%	0.8%	8.3%	0.0%	4.4%
**aspects**	Inconvenience	1.6%	2.4%	8.3%	0.9%	4.4%
**when**	Device dependency	25.8%	26.8%	13.9%	8.3%	10.9%
**acceptance**	Familiarity	4.7%	1.6%	2.8%	0.9%	10.9%
**in both contexts**	Reliability or security	1.6%	4.9%	5.6%	15.7%	13.0%
	Setup requirements	7.8%	4.9%	11.1%	7.4%	13.0%
	Speed	7.8%	1.6%	0.0%	2.8%	4.4%
	Nothing negative	28.1%	28.5%	25.0%	34.3%	17.4%
	Other	10.2%	13.0%	11.1%	16.7%	8.7%
	Not sure	10.2%	15.5%	13.9%	13.0%	13.0%
	Total of stated reasons	128	123	36	108	46
	N	110	121	31	115	37

Percentages represent the share of the total number of stated reasons. N: Number of participants providing rationale.

**One-time passcode (i):** For most respondents (89.4%, 95% CI [84.8%, 94.0%], N 169) one-time passcode was very or extremely familiar (Table 3). OTP was commonly in use: approximately half (51.5%, 95% CI [44.0%, 59.0%], N 169) of the participants used OTP frequently or 5–7 days a week. The majority (66.5%, 95% CI [59.4%, 73.6%], N 170) were somewhat to very happy to use OTP for account login, and an even higher proportion (76.5%, 95% CI [70.1%, 82.9%], N 170) were somewhat to very happy to use OTP for payment confirmation (Table 3). **Reliability/ security** was the clearly most frequent reason for acceptance, followed by distance by convenience and ease of use, in both situations (Table 4). The most common reason for rejecting OTP was **device dependency** in case of login purposes, and **reliability/ security** in case of payment confirmation. A subgroup of 105 participants (N 170) were happy to use OTP for both account login and payment confirmation, and when asked about any negative aspects, most commonly the participants did not see anything negative or problematic about the method (36 participants; Table 4). For the others, the most common negative aspects stated were device dependency, slow speed, and setup requirements. On the other hand, there were 15 participants who were unhappy to use OTP either for login or for payment; for these participants, the most common positive aspects were familiarity, reliability, low dependency on the device, and ease of use (Table 4).

In the free-text answers, two main themes dominated:

**Security**, where aspects were raised regarding

- Benefits, through examples such as memory advantage, the information value of receiving an OTP, and the quick expiration time of an OTP:

*‘If it’s instead of a password (and not as 2FA), then it’s one less password to remember’ (P41)*

*‘Lets me know if someone else is trying to access the system.’ (P3)*

*‘The OTP […] can expire within some minutes.’ (P126)*

- Concerns around device possession:

*‘Not safe. The one-time password gets sent a device, which is assumed only the person can access. It wouldn’t be that hard to gain access to that device.’ (P50)*

*‘If someone has your phone and phone number, they will receive the passcode.’ (P71)*

*‘As safe as the linked device.’ (P73)*

- Importance of being used in combination with other methods:

*‘would be happy to use it, but with passwords, this can be a secondary authentication’ (P70)*

*‘a good second-line of defence in tandem with other security measures’ (P101)*

**Practical use issues**, such as inconvenience and trouble receiving the passcode:

*‘…OTP every time you want to purchase an item or use a service can become annoying as [..] can cause problems with having to flip from one app to another’ (P69)*

*‘Not practical if phone has run out of battery’ (P102)*

*‘Sometimes, OTPs could be delayed. Source of OTP may not be verified as well.’ (P186)*

*‘only issue when mobile reception isn’t good’ (P95)*

*‘I would like to be able to copy and paste. If I am in an area of poor reception the code can take a long time to arrive. If I then ask for it to be resent, it can become muddled if the old passcode has [expired]. It just seems like another layer of bureaucracy on top of a password.’ (P43)*

*‘Works well if you’re using your phone to login and it automatically picks up the code, but it can be awkward to use if the phone doesn’t pick up the code or the site is being accessed using a different device.’ (P102)*

**Fingerprint (ii):** Similarly to OTP, fingerprint was a familiar method to the respondents, with 80.1% (95% CI [73.7%, 86.5%], N 151) of the respondents finding it very or extremely familiar (Table 3). Fingerprint was nearly as commonly used as OTP, with 50.7% (95% CI [42.70%, 58.70%], N 150) of participants using it frequently or even 5–7 days a week. Fingerprint was well accepted for account login, with the majority (78.8%, 95% CI [72.28%, 85.32%], N 151) somewhat to very happy to use it for this purpose. For payment confirmation, the ratio was similar, with 76.2% (95% CI [69.41%, 82.99%], N 151) somewhat to very happy to use fingerprint authentication (Table 3). **Convenience** was the most common reason for acceptance, followed by **reliability/ security** and **speed**, in both situations (Table 4). The most common reason for rejecting fingerprint was, by far, **reliability/ security**, followed by **device dependency**, in both purposes of use. A subgroup of 107 participants (N 151) were happy to use fingerprint for both account login and payment confirmation, and 35 of these did not really see anything negative about the method (Table 4). For the rest, the most frequent negative feature was dependency on the device. Meanwhile, among the 12 participants who would be unhappy to use fingerprint either for login or payment purposes, reliability was the main positive aspect (Table 4).

In the free-text answers, three main themes emerged, somewhat in line with OTP:

**Security**, with aspects on

- The advantage of uniqueness:

*‘No one else has my fingerprint.’ (P189)*

*‘This can be solely used by the owner as you can’t have same thumbprint’ (P19)*

- Concerns, with examples such as copying, hijacking, and implementation:

*‘Can be copied if a person isn’t aware of their surroundings.’ (P70)*

*‘someone could be forced or duped into confirming a transaction, e.g., when drunk or asleep’ (P40)*

*‘What is stopping digital signature hijacking from a device?’ (P56)*

*‘if your fingerprints are stolen this is not something the user has control over changing’ (P23)*

*‘Implementation can be poorly executed whilst appearing the same, not easily determined if this is the case’ (P29)*

**Convenience**, such as availability and relief from remembering details:

*‘Always available with the user.’ (P106)*

*‘When it works, it works well’ (P102)*

*‘More secure and simple to use’ (P82)*

*‘No need to remember the password.’ (P41)*

*‘No need to memorise any code, the pattern is constant.’ (P106)*

**Reliability, accuracy, and adaptability concerns**, such as correct reading of biometrics and options to use other fingers:

*‘My father and I can unlock each other’s biometrics, and I am not sure that fingerprints differentiate as much as they are claimed to do’ (P56)*

*‘others [..] have been frustrated if the device doesn’t read their fingerprint properly’ (P196)*

*‘sometimes as the device may find it difficult to read finger prints some of the time due to sweaty, oily and dusty fingers or device surface’ (P114)*

*‘my smartphones sensor is somewhat unreliable so can be frustrating’ (P99)*

*‘Dependent on one’s finger which sometimes may not readable by the device due to ageing’ (P37)*

*‘The system in using finger must be adaptive to recognise other fingers as well. If finger is cut, dry skin, cracked.’ (P27)*

**Voice recognition (iii):** Voice recognition was not widely known: Only about a fifth (22.2%, 95% CI [15.53%, 28.87%], N 149) of the respondents were very or extremely familiar with it (Table 3). Accordingly, voice recognition was rarely used: only 4.7% (95% CI [1.30%, 8.10%], N 149) of the participants used it frequently or even 5–7 days a week, while the majority (60.4%, 95% CI [52.55%, 68.25%]) said they had never used the method. Voice recognition was not popular for account login, with only 22.2% (95% CI [15.53%, 28.87%], N 149) somewhat to very happy to use it. For payment confirmation, the proportion was even lower than for login: 18.8% (95% CI [12.53%, 25.07%], N 149) (Table 3). In both cases, **convenience** was the most commonly stated reason for acceptance, followed by **reliability/ security** and **speed** (Table 4). The most common reason for rejecting voice recognition was, by far, **reliability/ security,** in both situations. A subgroup of 27 participants (N 149) were happy to use voice recognition for both login and payment confirmations, and 9 of these did not see anything negative about the method (Table 4). For the others, the most frequent negative aspects were dependency on the device and too high set-up requirements. A similar-sized subgroup of 26 participants would not accept voice recognition for either account login or payment confirmation purposes; however, they mentioned most commonly convenience, speed, and ease of use as positive aspects (Table 4).

In the free-text answers, three main themes emerged, much in line with fingerprint:

**Security issues**, with concerns such as imitation, hacking, and storage of biometric information:

*‘Not a secure method, a quality recording and other programmes can imitate your voice.’ (P70)*

*‘Storage of biometric info by other party seems inherent in most implementations.’ (P29)*

*‘My voice could be faked with AI, so not secure.’ (P7)*

*‘Voice can be hacked, so this method is not 100% reliable’ (P189)*

**Convenience and suitability issues**, with aspects such as effort and public places:

*‘Don’t want to talk, too much effort required from user.’ (P116)*

*‘Biased. Not suitable for people with speaking disability.’ (P39)*

*‘Embarrassing’ (P30)*

*‘Would feel conspicuous if out in public.’ (P36)*

*‘If I was shopping in a cafe, I don’t want to be saying [..] phrases into my phone’ (P102)*

*‘it’s not always convenient to speak out loud, such as in an office or busy place’ (P196)*

**Reliability, accuracy, and adaptability concerns**, with examples such as change in voice, transmission clarity, and siblings:

*‘My voice may change due a number of factors, and this would mean I don’t have access.’ (P110)*

*‘Factors such as sickness, aging, and mood can affect somebody’s voice.’ (P106)*

*‘I’m not convinced the technology is reliable enough to always distinguish the account holder or if transmission is always clear enough (my mobile connection is quite poor at home).’ (P40)*

*‘It seems not able to clearly distinguish siblings’ voices.’ (P6)*

*‘I wonder if voice is very unique, how probable it is for many people to overlap in voice’ (P73)*

Finally, a point to note is that two participants appeared to consider voice recognition with spoken passwords, although these were not indicated, which influenced their perceptions:

*‘Easily overheard and replicated.’ (P164)*

*‘Other persons may hear you say the password. Cannot be kept secret.’ (P108)*

**PIN code (iv):** Most participants (93%, 95%CI [88.82%, 97.18%], N 143) were very or extremely familiar with PIN authentication (Table 3). Accordingly, use of PIN code was common: majority (73.1%, 95%CI [65.78%, 80.42%], N 141) of the participants used it frequently or even 5–7 days a week. PIN code was well accepted for account login, with the majority (72.7%, 95%CI [65.40%, 80.00%], N 143) somewhat to very happy to use it for this purpose. For payment confirmation, the proportion was slightly higher than for logins, 74.1% (95% CI[66.92%, 81.28%], N 143) (Table 3). In both situations, **convenience** was the most frequent reason for acceptance of the method, followed by **speed** and **ease of use** in the case of account login and ease of use and **familiarity** in the case of payment confirmation (Table 4). The most common reason for rejecting PIN code was overwhelmingly **reliability/ security**, for both purposes. The majority (98) of the participants (N 143) were happy to use PIN code for both login and payments. While 37 of them stated that they didn’t really see anything negative or problematic about the method, for the rest the most common negative aspect was reliability/ security, followed by device dependency (Table 4). The seven participants who were unhappy to use PIN code for either login or payments did not state many positives about the method, with none of the options gaining more than two mentions (Table 4).

In the free-text answers, two main themes emerged, somewhat similarly to OTP:

**Security**, where the raised aspects included

- Requirement for adequate complexity and change:

*‘Its lack of complexity is a security risk. Also, it’s static, it doesn’t change as the OTP.’ (P41)*

- Requirement for combined approach:

*‘When used in conjunction with something you have [...] it seems to be convenient and secure.’ (P29)*

*‘Good if used in conjunction with other levels of authentication.’ (P70)*

*‘happy to use a PIN as a secondary action, such as accessing an app which I’ve logged onto previously, or using a card machine where the card needs to be present’ (P196)*

- Risk that the PIN is compromised:

*‘Someone might know the [PIN].’ (P131)*

*‘May be manipulated.’ (P124)*

*‘Can be poorly implemented using device pin that may be shared on a shared device etc.’ (P29)*

*‘PIN can be compromised and used by a third party or stolen.’ (P16)*

*‘Key loggers are used to access devices and steal PINs.’ (P6)*

*‘could be easy to guess; people tend to use numbers with significance’ (P99)*

**Practical use issues**, where the aspects included

- Benefits such as convenience and speed:

*‘For people that remember their pin numbers [it is] convenient and fast.’ (P38)*

*‘It is simple to use and set up’ (P180)*

- Concerns, with memorisation requirements particularly prominent:

*‘Have to remember different PINs. Keeping same PIN for every account is insecure.’ (P39)*

*‘Not too reliable because I can easily forget the pin’ (P189)*

*‘Easy to forget unless written down which compromises the security of the pin.’ (P108)*

*‘These PINs can be forgotten [and] misplaced when one has one or more account with different PINs.’ (P172)*

*‘Generally, relies on memory which may be problematic for some people.’ (P164)*

*‘As with passwords - it’s easy to forget, especially if not used in a while and as we shouldn’t have the same PIN for all accounts it’s easy to get muddled.’ (P40)*

**Finger swipe (v):** Less than half (38.7%, 95% CI [30.69%, 46.71%], N 142) of the participants were very or extremely familiar with finger swipe authentication (Table 3). Indeed, finger swipe was not commonly in use: only 21.1% (95% CI [14.39%, 27.81%], N 142) of the participants used it frequently or 5–7 days a week. A considerable proportion of participants (38.7%, 95% CI [30.69%, 46.71%], N 142) said they had never used the method. Finger swipe was not well accepted for account login, with only 31.0% (95% CI [23.39%, 38.61%], N 142) somewhat to very happy to use it for this purpose. For payment confirmation, the proportion was even lower, 24.0% (95% CI [16.98%, 31.02%], N 142) (Table 3). For both cases, the most common reason for acceptance was **convenience** (Table 4). The most common reason for rejecting finger swipe was, by far, **reliability/ security**, for both purposes. A subgroup of 33 participants (N 142) were happy to use finger swipe for both account login and payment confirmation. While eight participants saw nothing negative about finger swipe, the most common negative aspects stated by the others were reliability/ security issues and setup requirements (Table 4). Meanwhile, 54 participants were unhappy to use finger swipe for either account login or payment confirmation, but mentioned, most frequently, ease of use, speed, and convenience as positive aspects (Table 4).

In the free-text answers, two main themes dominated, in line with OTP and PIN code:

**Security**, particularly regarding the risk of copying:

*‘lost device = anyone could swipe’ (P164)*

*‘For me it is insecure.’ (P70)*

*‘It feels like it’s easy for someone shoulder surfing to pick it up.’ (P41)*

*‘This method requires you to cover up the swiping process for protection’ (P37)*

*‘Seems too easy to guess, are there that many possibilities so ensure brute forcing is not worth it? Also, shoulder-surfing would be easier, with pins you could potentially hide your inputs easier’ (P99)*

*‘Swipe can be done by anyone and it doesn’t hold much security’ (P3)*

**Practical use issues**, such as errors, cues for use, device requirements, and memorisation:

*‘Too open for error - i.e., swiping by mistake’ (P164)*

*‘It depends on the background being swiped over - if swiping over a pattern (e.g., dots) that is repeatable then it would be easy enough, but not sure how reliable or repeatable an unconstrained swipe would be.’ (P196)*

*‘When there is a broken screen it may be hard to draw such patterns’ (P59)*

*‘it would exclude users without access to a smart phone’ (P36)*

*‘It would need a touch device to work, so no good for many computers.’ (P196)*

*‘Can easily be forgotten.’ (P16)*

*‘If one forgets the pattern it becomes difficult to login’ (P38)*

Notably, two users appeared to consider the possibility of finger swipe as a method of recognising not only a simple pattern but instead, complex patterns and handwriting:

*‘method appears to be based on recognition of the handwriting of a user’ (P118)*

*‘It is a unique pattern set up by the user and so difficult to replicate by another and so seems secure’ (P114)*

#### Comparisons between authentication methods.

Differences between the methods were tested using the Friedman test, with paired-samples sign test post hoc analysis and Bonferroni adjustment (p = .005 with ten comparison pairs (five methods) and p = .0033 with 15 comparison pairs (five methods and AoC)). An exception was formed by the two cases of very few data points in the preferred methods comparisons, where binomial distribution was used instead of sign test, with adjusted p = .0033.

**Fig 1:**
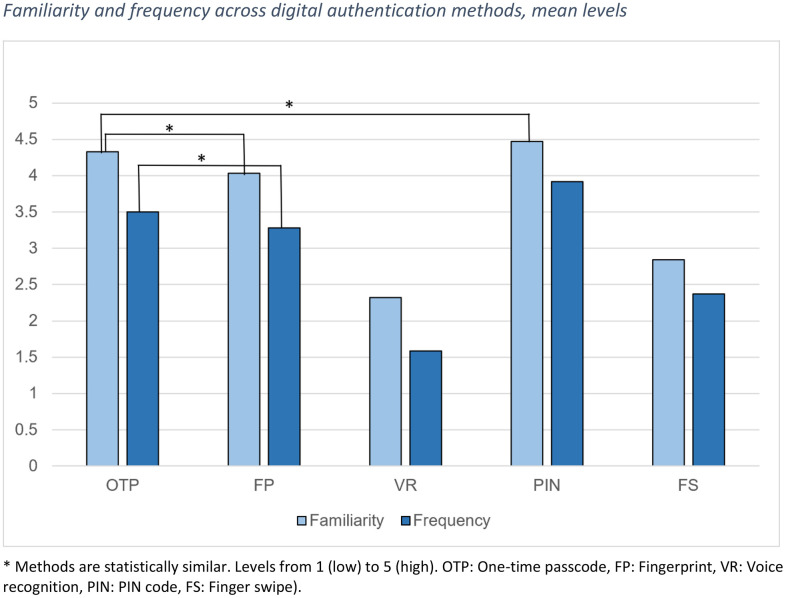
Familiarity and frequency across digital authentication methods, mean levels.

**Fig 2:**
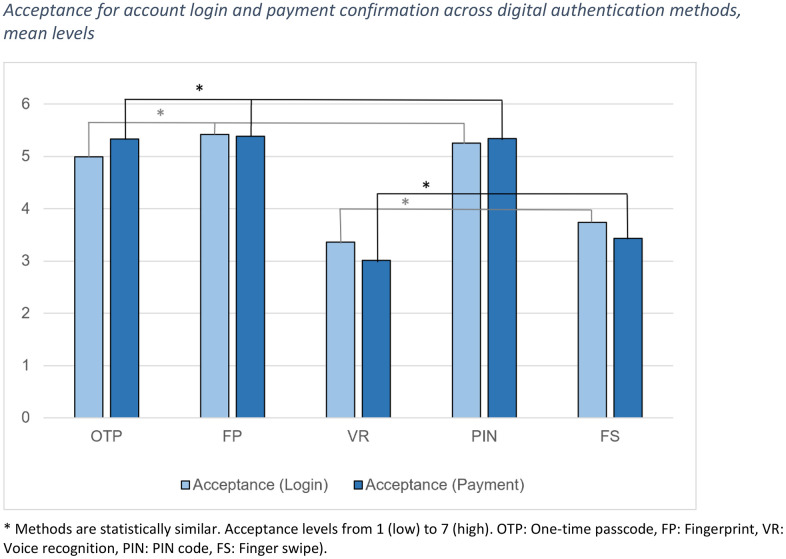
Acceptance for account login and payment confirmation across digital authentication methods, mean levels.

**Comparison between independent assessments:** While significant differences were found across the methods in each aspect of comparison, some methods were statistically similar to each other; these are marked with letters *a* and *b* in Table 3 and with asterisk (*) in Figs 1 and 2. For all other comparisons, the methods were significantly different from each other.

Familiarity: Familiarity with the authentication methods differed significantly between the methods (χ^2^(4) = 289.303, *p* < .001, N 141). This applied to all pairs of comparison except for OTP and fingerprint, and OTP and PIN code, suggesting that OTP was statistically equally familiar with fingerprint and with PIN code (OTP-FP *Z* = 2.697, *p* = .007; OTP-PIN *Z* = 2.121, *p* = .034; adjusted significance level.005) (Fig 1). Since PIN code was significantly more familiar than fingerprint, the most familiar methods were (shared first place) PIN and OTP, followed by fingerprint, finger swipe, and voice recognition.

Frequency of use: Frequency of use differed significantly between the methods (χ^2^(4)=258.283, *p* < .001, N 124). This applied to all method pairs except for OTP and fingerprint, which were statistically in equally frequent use (OTP-FP *Z* = .429, *p* = .668; adjusted significance level.005) (Fig 1). The most frequently used method was PIN code, followed by (shared second place) OTP and fingerprint, and then by finger swipe and voice recognition.

Acceptance for account login: Acceptance for login differed significantly between the methods (χ^2^(4)=183.895, *p* < .001, N 142). This applied to all pairs of comparison except four, which formed a divided pattern: One-time passcode, fingerprint, and PIN formed a cluster of methods that were statistically equally well accepted for account login (OTP-FP *Z* = 2.525, *p* = .012; OTP-PIN *Z* = 1.218, *p* = .223; FP-PIN *Z* = 1.735, *p* = .083), while they differed significantly from the second cluster of statistically similar voice recognition and finger swipe (VR-FS *Z* = 2.052, *p* = .040) (adjusted significance level.005) (Fig 2). Therefore, with slight differences in mean levels, fingerprint, PIN, and OTP shared the first place, while finger swipe and voice recognition shared the last place.

Acceptance for payment confirmation: Acceptance for payment differed significantly between the methods (χ^2^(4)=247.299, *p* < .001, N 142). Post-hoc tests showed the same clusters as in acceptance for account login: The statistically similar OTP, fingerprint, and PIN (OTP-FP *Z* = .105, *p* = .916; OTP-PIN *Z* = .406, *p* = .685; FP-PIN *Z* = .995, *p* = .320) differed significantly from the statistically similar voice recognition and finger swipe (VR-FS *Z* = 2.696, *p* = .007) (adjusted significance level.005) (Fig 2). As with acceptance for login, fingerprint, PIN, and OTP shared the first place and finger swipe and voice recognition were the least accepted methods.

**Comparison of ranking and preferences:** The rankings and preferences regarding the evaluated methods and the option of authentication of choice are presented in Table 5. For ranking, the levels varied from 1 (first) to 5 (last), and for preferences, the response was either selection (1) or non-selection (0); for comparison, the values on scale 0–1 are also shown on scale 1–5. The results are explored below.

**Table 5:** Rankings of the authentication methods including authentication of choice, for perceived ease of use and perceived security.

Aspect	Measure	One-time passcode	Finger- print	Voice recognition	PIN code	Finger swipe	AoC
Ranking*	Median	3	2	4	2	4	–
**from 1**^**st**^ **to 5**^**th**^ **place:**	Mean	2.7 ± 0.2^a^	1.9 ± 0.2	4.1 ± 0.2^b^	2.3 ± 0.2^a^	3.7 ± 0.2^b^	–
**perceived ease of use**	SD	1.30	1.05	1.10	1.10	1.24	–
	N	131	131	131	131	131	–
Ranking*	Median	2	2	4	3	4	–
**from 1st to 5th place:**	Mean	2.2 ± 0.2^a,b^	1.9 ± 0.2^a^	3.8 ± 0.2^c^	2.7 ± 0.2^b^	4.2 ± 0.2^c^	–
**perceived security**	SD	1.17	1.00	1.14	1.21	0.93	–
	N	129	129	129	129	129	–
**Preference: login**	Votes	69	78	10	62	15	41
(Total votes 275)	Share of all votes	25.1%	28.4%	3.6%	22.5%	5.5%	14.9%
**	Proportion of N	48.9% ± 8.3%	55.3% ± 8.2%	7.1% ± 4.2%	44% ± 8.2%	10.6% ± 5.1%	29.1% ± 7.5%
	Mean (scale 0–1)	0.49	0.55	0.07	0.44	0.11	0.29
	SD (scale 0–1)	0.50	0.50	0.26	0.50	0.31	0.46
	Mean (scale 1–5)	2.4 ± 0.4^a^	2.8 ± 0.4^a^	0.4 ± 0.2^c^	2.2 ± 0.4^a,b^	0.5 ± 0.3^c^	1.5 ± 0.4^b^
	SD (scale 1–5)	2.51	2.49	1.29	2.49	1.55	2.28
	N	141	141	141	141	141	141
**Preference: payments**	Votes	79	76	8	69	11	30
(Total votes 273)	Share of all votes	28.9%	27.8%	2.9%	25.3%	4.0%	11.0%
**	Proportion of N	56% ± 8.2%	53.9% ± 8.2%	5.7% ± 3.8%	48.9% ± 8.3%	7.8% ± 4.4%	21.3% ± 6.8%
	Mean (scale 0–1)	0.56	0.54	0.06	0.49	0.08	0.21
	SD (scale 0–1)	0.50	0.50	0.23	0.50	0.27	0.41
	Mean (scale 1–5)	2.8 ± 0.4^a^	2.7 ± 0.4^a^	0.3 ± 0.2^c^	2.4 ± 0.4^a^	0.4 ± 0.2^b,c^	1.1 ± 0.3^b^
	SD (scale 1–5)	2.49	2.50	1.16	2.51	1.35	2.05
	N	141	141	141	141	141	141

AoC: Authentication of Choice: Selection of method at the point of authentication.

* Ranking: Higher value = lower regard; highest = 1, lowest = 5; shared rankings and non-rankings accepted. ** Rating: Higher value = higher regard.

^a^, ^b^, ^c^: Methods sharing the same letter are statistically similar. Margins of error at 95% confidence level.

**Fig 3:**
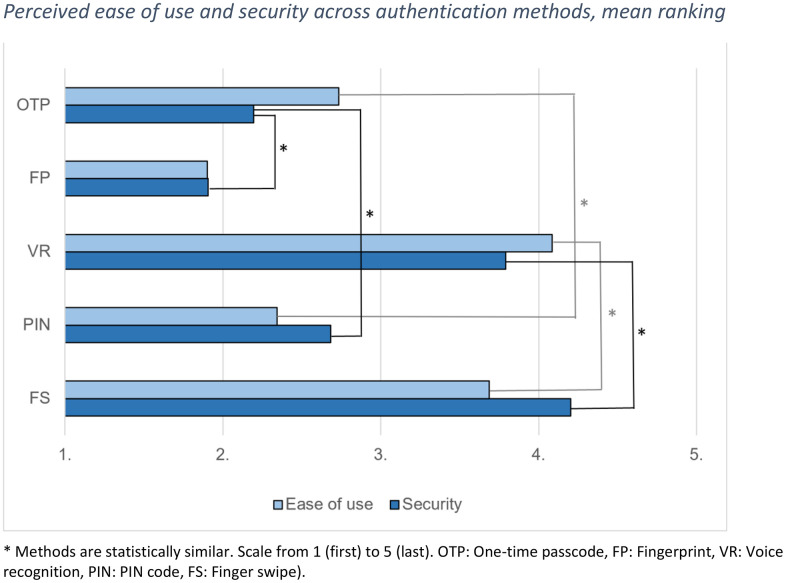
Perceived ease of use and security across authentication methods, mean ranking.

Ranking for ease of use: Participants’ rankings for ease of use differed significantly between the methods (χ^2^(4) = 183.676, *p* < .001, N 131). Post-hoc tests showed that this applied to all pairs of comparison except for OTP and PIN, which were ranked equally high, and voice recognition and finger swipe, which were ranked equally low (OTP-PIN *Z* = 2.543, *p* = .011; VR-FS *Z* = 2.289, *p* = .022; adjusted significance level.005). The highest ranked was fingerprint, followed by PIN and OTP in a shared second place. Finger swipe and voice recognition shared the last place (Fig 3).

Ranking for security: Participants’ rankings for security differed significantly between the methods (χ^2^(4) = 215.424, *p* < .001, N 129). Post-hoc tests showed that this applied to all pairs of comparison except for OTP and fingerprint, OTP and PIN code, and voice recognition and finger swipe, suggesting that OTP was perceived as equally secure as fingerprint and PIN code, and voice recognition and finger swipe were perceived as equally low in security aspects (OTP-FP *Z* = 0.880, *p* = .379; OTP-PIN *Z* = 2.662, *p* = .008; VR-FS *Z* = 1.871, *p* = .061; adjusted significance level.005) (Fig 3). Since fingerprint was ranked significantly higher than PIN code, the first place was shared by fingerprint and OTP, followed by PIN code, with voice recognition and finger swipe sharing the last place (Fig 3).

**Fig 4:**
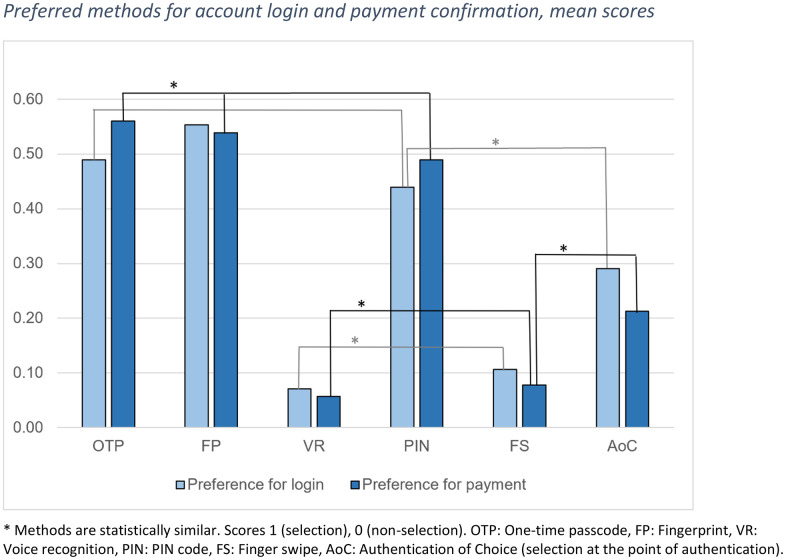
Preferred methods for account login and payment confirmation, mean scores.

Preferred methods for account login: When participants were asked to select their favourite methods for account login, there was a significant difference between the favourability of the different methods (χ^2^(5) = 129.868, *p* < .001, N 141). Post-hoc tests showed that this applied to all pairs of comparison except five. Four of these formed a divided pattern in line with the individual assessments of acceptance for account login tested earlier: One-time passcode, fingerprint, and PIN formed a cluster of methods that were statistically equal favourites for account login (OTP-FP *Z* = 0.963, *p* = .336; OTP-PIN *Z* = 0.702, *p* = .483; FP-PIN *Z* = 1.905, *p* = .057), while they differed significantly from a second cluster of statistically similar voice recognition and finger swipe (VR-FS *p* = .227, exact, 2-tailed, binomial distribution) (adjusted significance level.0033) (Fig 4). Meanwhile, the fifth pair, PIN and AoC were equally favoured for account login (PIN-AoC *Z* = 2.341, *p* = .019, adjusted significance level.0033). Therefore, with slight differences in mean levels, fingerprint, OTP, and PIN shared the first place, followed by AoC, while finger swipe and voice recognition gained very few votes and shared the last place (Fig 4).

Preferred methods for payment confirmation: When participants were asked to select their favourite methods for payment confirmation, there was a significant difference between the favourability of the different methods (χ^2^(5) = 173.480, *p* < .001, N 141). Post-hoc tests showed that this applied to all comparison pairs except five, where four were the same as for preference for account login and for acceptance for payment confirmation tested earlier: One-time passcode, fingerprint, and PIN formed a cluster of methods that were statistically equal favourites for payment confirmation (OTP-FP *Z* = 0.237, *p* = .812; OTP-PIN *Z* = 1.108, *p* = .268; FP-PIN *Z* = 0.756, *p* = .450), while, again, they differed significantly from the second cluster of statistically similar voice recognition and finger swipe (VR-FS *p* = .581, exact, 2-tailed, binomial distribution) (adjusted significance level.0033) (Fig 4). Meanwhile, the fifth pair was formed by FS and AoC, which were equally favoured for payment confirmation (FS-AoC *Z* = 2.882, *p* = .004, adjusted significance level.0033). Similar to the favourites for account login purposes, OTP, fingerprint, and PIN shared the first place, followed by AoC, while finger swipe and voice recognition gained very few votes and shared the last place (Fig 4).

### Stage 2: Associations

The relationships between the aspects in the ordinal data were tested using Spearman rank-order correlation coefficient; the results are presented in Table 6 and explored below.

**Table 6:** Correlations of the reported and evaluated aspects across the authentication methods.

Assessment	OTP					FP					VR				
	rho	p	95% CI			rho	p	95% CI			rho	p	95% CI		
			Lower	Upper	N			Lower	Upper	N			Lower	Upper	N
Familiarity- Frequency	0.332	0.000	0.183	0.465	163	0.603	0.000	0.484	0.700	145	0.647	0.000	0.538	0.736	144
Familiarity – Acceptance (login)	0.005	0.948	−0.150	0.160	169	0.205	0.012	0.042	0.357	151	0.013	0.876	−0.153	0.178	149
Familiarity – Acceptance (payment)	0.164	0.033	0.009	0.311	169	0.234	0.004	0.073	0.384	151	0.022	0.787	−0.144	0.187	149
Familiarity – Ease of use*	0.009	0.921	−0.167	0.184	133	−0.317	0.000	−0.465	−0.151	135	−0.090	0.303	−0.261	0.087	133
Familiarity – Security*	−0.157	0.074	−0.326	0.020	130	−0.227	0.008	−0.387	−0.054	133	−0.069	0.436	−0.242	0.109	131
Familiarity – Preference (login)	−0.069	0.416	−0.237	0.103	140	0.010	0.903	−0.160	0.180	141	0.143	0.090	−0.028	0.306	141
Familiarity- Preference (payment)	−0.029	0.735	−0.199	0.143	140	0.066	0.439	−0.106	0.233	141	0.190	0.024	0.021	0.349	141
Familiarity – gender	0.029	0.739	−0.145	0.201	136	−0.118	0.168	−0.285	0.055	137	−0.159	0.064	−0.323	0.014	137
Familiarity – age	−0.054	0.529	−0.224	0.119	138	−0.214	0.011	−0.372	−0.045	139	−0.034	0.688	−0.204	0.138	139
Familiarity – IT use	0.242	0.004	0.073	0.397	138	0.190	0.025	0.020	0.350	139	0.086	0.316	−0.087	0.253	139
Frequency – Acceptance (login)	0.041	0.601	−0.118	0.198	163	0.169	0.042	0.002	0.328	145	0.150	0.073	−0.019	0.310	144
Frequency – Acceptance (payment)	0.105	0.184	−0.054	0.259	163	0.201	0.015	0.034	0.357	145	0.071	0.397	−0.098	0.237	144
Frequency – Ease of use*	0.026	0.768	−0.152	0.202	130	−0.178	0.041	−0.343	−0.002	132	−0.154	0.082	−0.323	0.025	129
Frequency – Security*	−0.030	0.740	−0.208	0.150	127	−0.058	0.509	−0.233	0.120	130	−0.082	0.356	−0.257	0.098	128
Frequency – Preference (login)	−0.006	0.945	−0.180	0.169	134	0.095	0.274	−0.080	0.264	135	0.073	0.401	−0.102	0.243	136
Frequency – Preference (payment)	0.024	0.781	−0.151	0.198	134	0.073	0.402	−0.102	0.244	135	0.046	0.593	−0.128	0.218	136
Frequency – gender	−0.029	0.742	−0.205	0.149	130	−0.105	0.231	−0.277	0.072	131	−0.082	0.346	−0.254	0.094	133
Frequency – age	0.094	0.283	−0.083	0.266	132	−0.221	0.010	−0.382	−0.048	133	−0.112	0.196	−0.280	0.063	135
Frequency – IT use	0.209	0.016	0.035	0.370	133	0.221	0.011	0.047	0.381	133	0.078	0.371	−0.098	0.249	134
Acceptance (login) – Acceptance (payment)	0.596	0.000	0.486	0.687	170	0.809	0.000	0.744	0.860	151	0.861	0.000	0.811	0.899	149
Acceptance (login) – Ease of use*	−0.274	0.001	−0.428	−0.104	133	−0.345	0.000	−0.489	−0.182	135	−0.254	0.003	−0.410	−0.082	133
Acceptance (login) – Security*	−0.144	0.101	−0.314	0.034	130	−0.313	0.000	−0.463	−0.146	133	−0.147	0.094	−0.315	0.030	131
Acceptance (login) – Preference (login)	0.225	0.007	0.057	0.381	141	0.240	0.004	0.073	0.394	141	0.298	0.000	0.134	0.445	141
Acceptance (login) – Preference (payment)	0.229	0.006	0.061	0.384	141	0.234	0.005	0.067	0.389	141	0.220	0.009	0.052	0.376	141
Acceptance (login) – gender	−0.086	0.318	−0.255	0.088	137	−0.097	0.262	−0.265	0.077	137	0.046	0.594	−0.128	0.217	137
Acceptance (login) – age	0.109	0.201	−0.063	0.275	139	−0.156	0.067	−0.319	0.016	139	−0.014	0.868	−0.185	0.158	139
Acceptance (login) – IT use	0.149	0.081	−0.023	0.312	139	0.186	0.028	0.015	0.346	139	0.044	0.607	−0.128	0.214	139
Acceptance (payment) – Ease of use*	−0.189	0.029	−0.353	−0.014	133	−0.310	0.000	−0.459	−0.144	135	−0.225	0.009	−0.385	−0.052	133
Acceptance (payment) – Security*	−0.195	0.026	−0.359	−0.018	130	−0.376	0.000	−0.517	−0.215	133	−0.144	0.100	−0.313	0.033	131
Acceptance (payment) – Preference (login)	0.269	0.001	0.104	0.420	141	0.270	0.001	0.105	0.421	141	0.318	0.000	0.156	0.463	141
Acceptance (payment) – Preference (payment)	0.324	0.000	0.162	0.468	141	0.370	0.000	0.213	0.508	141	0.290	0.000	0.127	0.439	141
Acceptance (payment) – gender	−0.052	0.547	−0.222	0.122	137	−0.185	0.031	−0.346	−0.013	137	0.035	0.686	−0.139	0.206	137
Acceptance (payment) – age	0.117	0.171	−0.056	0.283	139	−0.105	0.217	−0.272	0.067	139	0.054	0.529	−0.119	0.223	139
Acceptance (payment) – IT use	0.205	0.015	0.035	0.364	139	0.135	0.114	−0.038	0.299	139	0.032	0.706	−0.140	0.203	139
Ease of use* - Preference (login)	−0.318	0.000	−0.467	−0.150	132	−0.321	0.000	−0.469	−0.155	134	−0.173	0.047	−0.339	0.003	132
Ease of use* - Preference (payment)	−0.158	0.071	−0.324	0.019	132	−0.392	0.000	−0.530	−0.233	134	−0.160	0.067	−0.327	0.016	132
Ease of use* - gender	−0.022	0.807	−0.200	0.157	128	0.088	0.322	−0.091	0.261	130	0.040	0.652	−0.139	0.217	128
Ease of use* - age	−0.140	0.111	−0.310	0.038	130	0.089	0.309	−0.088	0.261	132	0.078	0.377	−0.100	0.252	130
Ease of use* - IT use	−0.028	0.754	−0.204	0.150	130	−0.068	0.439	−0.241	0.109	132	−0.026	0.765	−0.203	0.151	130
Security* - Preference (login)	−0.416	0.000	−0.553	−0.257	129	−0.394	0.000	−0.533	−0.234	132	−0.176	0.046	−0.342	0.002	130
Security* - Preference (payment)	−0.408	0.000	−0.546	−0.248	129	−0.418	0.000	−0.554	−0.262	132	−0.209	0.017	−0.372	−0.033	130
Security* - gender	−0.172	0.055	−0.342	0.009	125	0.161	0.070	−0.018	0.330	128	−0.188	0.035	−0.356	−0.009	126
Security* - age	0.005	0.952	−0.174	0.184	127	−0.001	0.991	−0.178	0.176	130	−0.054	0.547	−0.230	0.126	128
Security* - IT use	−0.173	0.052	−0.342	0.006	127	−0.067	0.446	−0.242	0.111	130	0.100	0.263	−0.080	0.273	128
Preference (login) – Preference (payment)	0.581	0.000	0.456	0.684	141	0.600	0.000	0.478	0.699	141	0.768	0.000	0.688	0.830	141
Preference (login) – gender	−0.092	0.286	−0.260	0.082	137	−0.237	0.005	−0.394	−0.068	137	−0.002	0.978	−0.175	0.170	137
Preference (login) – age	0.176	0.038	0.005	0.338	139	0.040	0.638	−0.132	0.210	139	0.001	0.990	−0.170	0.172	139
Preference (login) – IT use	0.096	0.262	−0.077	0.263	139	−0.066	0.442	−0.234	0.107	139	−0.029	0.739	−0.199	0.143	139
Preference (payment) – gender	−0.105	0.223	−0.272	0.069	137	−0.135	0.116	−0.301	0.038	137	0.062	0.473	−0.112	0.232	137
Preference (payment) – age	0.110	0.198	−0.063	0.276	139	0.102	0.230	−0.070	0.269	139	0.044	0.603	−0.128	0.214	139
Preference (payment) – IT use	0.218	0.010	0.049	0.375	139	−0.139	0.102	−0.303	0.033	139	−0.176	0.038	−0.337	−0.005	139
**Assessment**	**PIN**					**FS**					**AoC**				
	**rho**	**p**	**95% CI**			**rho**	**p**	**95% CI**			**rho**	**p**	**95% CI**		
			**Lower**	**Upper**	**N**			**Lower**	**Upper**	**N**			**Lower**	**Upper**	**N**
Familiarity- Frequency	0.360	0.000	0.202	0.501	139	0.776	0.000	0.696	0.837	135					
Familiarity – Acceptance (login)	0.203	0.015	0.036	0.360	143	0.193	0.021	0.024	0.351	142					
Familiarity – Acceptance (payment)	0.202	0.015	0.035	0.359	143	0.033	0.694	−0.137	0.202	142					
Familiarity – Ease of use*	−0.031	0.726	−0.205	0.145	133	−0.247	0.004	−0.405	−0.075	133					
Familiarity – Security*	0.026	0.762	−0.149	0.201	133	0.030	0.739	−0.149	0.206	129					
Familiarity – Preference (login)	−0.003	0.976	−0.173	0.168	141	0.064	0.448	−0.107	0.232	141					
Familiarity- Preference (payment)	0.003	0.974	−0.167	0.173	141	0.041	0.629	−0.130	0.210	141					
Familiarity – gender	−0.038	0.657	−0.209	0.135	137	−0.097	0.260	−0.265	0.077	137					
Familiarity – age	0.055	0.516	−0.117	0.225	139	−0.163	0.055	−0.325	0.009	139					
Familiarity - ITuse	0.191	0.024	0.020	0.351	139	0.147	0.085	−0.025	0.310	139					
Frequency – Acceptance (login)	0.180	0.034	0.009	0.341	139	0.335	0.000	0.171	0.480	135					
Frequency – Acceptance (payment)	0.185	0.029	0.014	0.345	139	0.136	0.115	−0.038	0.303	135					
Frequency – Ease of use*	−0.043	0.622	−0.217	0.133	133	−0.138	0.121	−0.310	0.042	127					
Frequency – Security*	−0.030	0.733	−0.204	0.146	133	0.116	0.203	−0.068	0.292	123					
Frequency – Preference (login)	0.029	0.734	−0.144	0.200	138	0.140	0.106	−0.035	0.307	134					
Frequency – Preference (payment)	0.066	0.443	−0.107	0.235	138	0.040	0.649	−0.136	0.213	134					
Frequency – gender	0.028	0.747	−0.147	0.202	134	−0.023	0.792	−0.200	0.154	130					
Frequency – age	−0.002	0.984	−0.175	0.172	136	−0.091	0.300	−0.263	0.086	132					
Frequency - ITuse	0.123	0.155	−0.052	0.290	136	0.098	0.263	−0.079	0.269	132					
Acceptance (login) – Acceptance (payment)	0.846	0.000	0.789	0.888	143	0.843	0.000	0.786	0.886	142					
Acceptance (login) – Ease of use*	−0.184	0.034	−0.348	−0.010	133	−0.138	0.113	−0.306	0.038	133					
Acceptance (login) – Security*	−0.293	0.001	−0.445	−0.124	133	−0.262	0.003	−0.420	−0.088	129					
Acceptance (login) – Preference (login)	0.182	0.030	0.013	0.342	141	0.281	0.001	0.116	0.430	141					
Acceptance (login) – Preference (payment)	0.176	0.036	0.006	0.336	141	0.150	0.076	−0.021	0.312	141					
Acceptance (login) – gender	−0.115	0.180	−0.282	0.059	137	0.128	0.137	−0.046	0.294	137					
Acceptance (login) – age	−0.117	0.172	−0.282	0.056	139	−0.031	0.716	−0.201	0.141	139					
Acceptance (login) - ITuse	−0.039	0.646	−0.209	0.133	139	−0.018	0.831	−0.189	0.154	139					
Acceptance (payment) – Ease of use*	−0.172	0.048	−0.337	0.003	133	−0.098	0.263	−0.268	0.079	133					
Acceptance (payment) – Security*	−0.422	0.000	−0.556	−0.266	133	−0.324	0.000	−0.475	−0.155	129					
Acceptance (payment) – Preference (login)	0.273	0.001	0.108	0.424	141	0.218	0.009	0.050	0.374	141					
Acceptance (payment) – Preference (payment)	0.267	0.001	0.101	0.418	141	0.143	0.092	−0.028	0.305	141					
Acceptance (payment) – gender	−0.130	0.130	−0.296	0.044	137	0.094	0.275	−0.080	0.262	137					
Acceptance (payment) – age	−0.156	0.066	−0.319	0.016	139	0.005	0.953	−0.166	0.176	139					
Acceptance (payment) - ITuse	−0.108	0.206	−0.274	0.065	139	−0.050	0.557	−0.220	0.122	139					
Ease of use* - Preference (login)	−0.215	0.013	−0.376	−0.040	132	−0.106	0.228	−0.276	0.072	132					
Ease of use* - Preference (payment)	−0.175	0.044	−0.341	0.001	132	−0.129	0.140	−0.298	0.048	132					
Ease of use* - gender	0.139	0.117	−0.040	0.310	128	−0.188	0.033	−0.355	−0.010	128					
Ease of use* - age	−0.117	0.185	−0.288	0.062	130	0.086	0.329	−0.092	0.259	130					
Ease of use* - ITuse	0.185	0.035	0.008	0.351	130	0.032	0.720	−0.146	0.208	130					
Security* - Preference (login)	−0.261	0.002	−0.418	−0.089	132	−0.169	0.057	−0.337	0.010	128					
Security* - Preference (payment)	−0.245	0.005	−0.403	−0.072	132	−0.119	0.181	−0.291	0.061	128					
Security* - gender	0.177	0.046	−0.002	0.344	128	−0.022	0.808	−0.203	0.160	124					
Security* - age	0.118	0.183	−0.061	0.289	130	0.009	0.916	−0.171	0.189	126					
Security* - ITuse	0.186	0.034	0.009	0.352	130	−0.035	0.697	−0.214	0.146	126					
Preference (login) – Preference (payment)	0.676	0.000	0.572	0.759	141	0.500	0.000	0.361	0.618	141	0.583	0.000	0.458	0.685	141
Preference (login) – gender	−0.137	0.110	−0.302	0.036	137	0.055	0.526	−0.119	0.225	137	0.226	0.008	0.055	0.383	137
Preference (login) – age	0.146	0.087	−0.026	0.309	139	−0.058	0.496	−0.227	0.114	139	0.018	0.829	−0.153	0.189	139
Preference (login) - ITuse	−0.120	0.160	−0.285	0.053	139	0.063	0.462	−0.110	0.232	139	−0.104	0.221	−0.271	0.068	139
Preference (payment) – gender	−0.186	0.029	−0.348	−0.014	137	−0.010	0.910	−0.182	0.163	137	0.147	0.086	−0.026	0.312	137
Preference (payment) – age	0.175	0.040	0.003	0.336	139	0.005	0.956	−0.167	0.176	139	0.120	0.160	−0.052	0.285	139
Preference (payment) - ITuse	−0.245	0.004	−0.400	−0.077	139	0.036	0.673	−0.136	0.206	139	−0.049	0.568	−0.218	0.123	139

95% CI estimation based on Fisher’s r-to-z transformation. Estimation of standard error based on the formula proposed by Fieller, Hartley, and Pearson. Green shading: p < 0.05. Blue shading: Assessment where p. < 05 for all authentication methods. Grey shading: For all authentication methods, p ≥ .05. Gender coding: 0: Male, 1: Female. * One ranking scale with inverted order (1 = first, 5 = last) ➔ Negative association indicates a link between an increase in perceived security/ ease of use and an increase in the comparison parameter.

#### Consistency of responses between sections of survey.

The association between *Familiarity* and *Frequency* was consistently significant and positive for all methods, with medium to large effect sizes (r = .332 ….776) and p values < .001 (Table 6), as could be expected, supporting the assumption of valid (non-random) data. Consistency was also seen between *Acceptance (login)* and *Preference (login),* with significant positive correlations for every method (effect sizes small, r = .182 ….298). Also the correlation *Acceptance (payment) – Preference (payment)* was significant (effect sizes small to medium r = .267 ….370) for all other methods except for finger swipe (p = .092). (For finger swipe, the tendency was of much higher regard for the method considering acceptance of it for payment (mean rating 3.43/ 5) than actual preference for it in payment situation (respective mean rating 0.39/ 5).) Overall, the results suggests that **the evaluations of the methods from two different stages of the survey, with different presentation order of the methods, were generally well aligned**. Combined with the fact that the levels of appreciation for the methods in Stage 1 did not follow the order of presentation in the first part of the survey, **the data support the suggestion that the evaluations are valid and the conclusions were not affected by the order of the presentation in the first section**.

#### Context (low or high risk).

The associations between login and payment evaluations, whether observing the exact same viewpoint *(Acceptance (login) – Acceptance (payment), Preference (login) – Preference (payment))* or across slightly different viewpoints *(Acceptance (login) – Preference (payment), Acceptance (payment) – Preference (login))* were significant (p < .05) in nearly all 21 cases, including the applicable test *Preference (login) – Preference (payment)* for AoC (Table 6). The only exception was *Acceptance (login) – Preference (payment)* in the case of finger swipe (p = .076), where the tendency was of much higher acceptance of finger swipe for login (mean rating 3.74/ 5) than of selection of it for payment (respective mean rating 0.39/ 5). Overall, in 20 out of 21 cases the evaluation of the method for login purposes correlated with its evaluation for payment confirmation. Effect sizes were large for the exact pairs *Acceptance (login) – Acceptance (payment)* and *Preference (login) – Preference (payment)* (r = .500 ….861) and small to medium across these two viewpoints (*Acceptance (login) – Preference (payment),* and *Acceptance (payment) – Preference (login))* (r = .176 ….318) (Table 6). Overall, the results suggest that **the level of risk in the authentication situation** – whether the method was suggested to be used in a lower risk context such as online shop account login, or in a higher risk context, such as payment confirmation – **was not significantly linked with participant’s choices.**

#### Perceived security.

Higher perceived security as such was a factor significantly associated with higher regard of the method in 15 out of 20 tests evaluating the relationship between *Security* and participants’ choices (*Acceptance (login)*, *Acceptance (payment)*, *Preference (login),* and *Preference (payment);* Table 6). The five cases where the association was not significant were for OTP, regarding *Acceptance (login)* (p = .101); voice recognition, regarding *Acceptance (login)* (p = .094) and *Acceptance (payment)* (p = .100); and finger swipe, regarding *Preference (login)* (p = .057) and *Preference (payment)* (p = .181). Effect sizes in the significant correlations across all methods varied from small to medium (|r| = .176 …. 418). Only for FP and PIN, all tests showed significant positive associations; for FP, these were all of medium effect size (|r| = .313 ….418), while for PIN they varied from small to medium (|r| = .245 … 422). For OTP, particularly the links between perceived security and actual selection of the method for login and payment were stronger (|r| = .416 and |r| = .408, respectively), while the association with *Acceptance (login*) was weaker (|r| = .195). For VR and FS the effect sizes of the two significant tests each were small, except for FS regarding the slightly stronger association with *Acceptance (payment)* (|r| = .324). The results would suggest that **higher perceived security was moderately linked with participants’ higher regard for FP, while the phenomenon was robust also for PIN, of varying strength for OTP, and weaker for VR and FS.**

#### Perceived ease of use.

Similarly to perceived security, higher perceived ease of use was significantly associated with higher regard for the method in the majority of the tests investigating participants’ choice: Fourteen out of the 20 tests evaluating the relationship between *Ease of use* and *Acceptance (login)*, *Acceptance (payment)*, *Preference (login),* and *Preference (payment)* across the methods showed significant results (Table 6). The method for which higher perception of *Ease of use* correlated significantly, and strongest, with all four aspects of choice was fingerprint, with medium effect sizes (|r| = 0.310…0.392, p < .001). The same significant correlations applied also in the case of PIN code, but the effect was weaker (|r| = .172 ….215). For OTP and VR, higher regard of *Ease of use* correlated with *Acceptance (login)*, *Acceptance (payment)*, and *Preference (login*), but not *Preference (payment).* These correlations were mainly of small effect size, except for *Preference (login*) for OTP, which was medium (|r| = .318, p < .001). Finally, an exceptional case was FS, for which there were no significant correlations between perceived *Ease of use* and participants’ choices. Overall, the results would suggest that **higher perception of** *Ease of use* **correlated moderately with higher regard for fingerprint, and, to a slightly lesser extent, for PIN code, followed by OTP and VR. For finger swipe,** *Ease of use* **did not correlate with participants’ choices**.

#### Prior experience.

Prior experience, as observed through *Familiarity* and *Frequency*, was associated to a varying extent with participants’ choices (*Acceptance (login), Acceptance (payment), Preference (login), Preference (payment)*) and perceptions (*Security, Ease of use*) depending on the method. Overall, significant correlations were not very frequent across the methods: only 16 of the 60 potential correlations were significant. The highest number of significant links was observed for fingerprint: A significant correlation was found in seven tests (*Familiarity* with *Acceptance (login)*, *Acceptance (payment)*, *Ease of use, Security*; and *Frequency* with *Acceptance (login)*, *Ease of use,* and *Security*) out of the 12 potential correlations for FP, with small to medium effect sizes (|r| = .169 ….317) (Table 6). For PIN code, both higher familiarity and higher frequency were weakly linked with higher acceptance for login and for payment (|r| = .180 ….203). For finger swipe, higher familiarity was linked with higher acceptance for login (r = .193) and higher perceived ease of use (|r| = .247); and higher frequency was, more strongly, linked with acceptance for login (r = .335). For OTP, higher familiarity was weakly linked with higher acceptance for payment (r = .164). For voice recognition, higher familiarity was linked with higher preference for payment (r = .190). Overall, the results suggest that **while significant correlations between experience and method evaluations were not frequent, they were positive, and more common with fingerprint than with the other methods.**

#### Experience on IT use.

IT expertise was occasionally (in seven cases out of 32 applicable tests) significantly associated with choices and perceptions, although the effect sizes were small. This occurred across OTP, FP, VR, and PIN, but never for FS or AoC. Specifically, for OTP, higher levels of IT expertise were weakly linked with higher regard of the method for payment confirmation, both in terms of *Acceptance (payment)* (r = .205) and *Preference (payment)* (r = .218*).* For FP, higher IT expertise was weakly linked with higher *Acceptance (login)* (r = .186). For VR, in turn, there was a negative association: higher IT expertise was weakly linked with lower *Preference (payment)* (|r| = .176). For PIN, the links were negative as well: higher IT expertise was weakly associated with lower regard for PIN considering *Preference (payment)*, |r| = .245), perceived *Ease of use* (|r| = .185) and perceived *Security* (|r| = .186). Finally, regarding the potential links between experience in IT and familiarity and frequency of use across the methods, the associations were few and weak: **higher** experience in IT was positively associated with *Familiarity* for OTP and FP (r = .190, r = .242, respectively) but not for VR and FS, and positively associated with *Frequency* only for OTP (r = .209) and FP (r = .221) (Table 6). **Overall, the correlations between expertise in IT and the participants’ responses were infrequent and weak, but mostly observed with PIN code** (three cases where IT use was linked with **lower** evaluations), followed by **OTP** (two cases, IT use linked with **higher** evaluations), **FP** (one case, IT use linked with **higher** evaluation), and **VR** (one case, IT use linked with **lower** evaluation). For finger swipe and AoC, IT use was not linked with lower or higher evaluations.

#### Gender.

**Gender was not frequently significantly associated with participants’ choices or perceptions, with some exceptions** for which the effect sizes were small. Considering fingerprint, females tended to rate *Acceptance (payment)* and *Preference (login)* slightly lower than males (|r| = .185, |r| = .237, respectively) (Table 6). For voice recognition, females rated *Security* slightly higher than males (r = .188). For PIN, females rated both *Security* and *Preference (payment)* slightly lower than males (r = .177, |r| = .186, respectively). For finger swipe, females rated *Ease of use* slightly higher than males (|r| = .188). Authentication of choice, in turn, was rated higher regarding *Preference (login)* by females than males (r = .226). Finally, gender was **not significantly associated with** *Familiarity* **or *Frequency*** for any method.

#### Age.

**Age was not significantly associated with choices and perceptions except in two cases with small effect sizes**. For OTP, *Acceptance (login)* was slightly higher at a higher age (r = .176), and for PIN code, *Preference (payment)* was slightly higher at a higher age (r = .175) (Table 6). In addition, for fingerprint, higher age was weakly linked with lower ***Frequency*** (|r| = .221).

## Discussion

The current study explored end-users’ views on a range of digital authentication approaches (OTP, FP, VR, PIN, FS, and AoC) in two different contexts (end-user account login and payment confirmation). Participants’ familiarity, frequency of use, acceptance, ranking for perceived security and ease of use, preferences, and perception of benefits and concerns regarding the different methods were investigated, along with their gender, age, and level of IT use.

### Evaluations of the authentication methods

#### Summary of results.

Three of the authentication methods – OTP, FP, and PIN– emerged as a cluster of clear favourites across measures as well as contexts. They outranked the second cluster formed by VR and FS, and preceded also AoC, which was placed between the two clusters in terms of preferred methods for account login and payment confirmation. Regarding perceived ease of use, FP was the favourite, followed by PIN and OTP. For perceived security, FP shared the first place with OTP, followed by PIN. In terms of preferred methods for account login and payment confirmation, FP shared the first place with both OTP and PIN. After these three favourites, in the applicable cases (preferences for account login and payment confirmation), AoC took the fourth place, although for preference for login purposes, it was statistically similar to PIN, and for payment, it was statistically similar to FS. Meanwhile, VR and FS were consistently the lowest rated methods and consistently statistically similar to each other. Across the methods, the comments and rationale the participants provided demonstrated a wide variation in their knowledge and perceptions on the specific characteristics of each method, particularly around convenience of use and security issues, influencing their choices.

#### Types of authentication.

Overall, the favourites ranged across all three presented types of authentication (biometrics, knowledge factors, and possession factors), which suggests that the application of any one of these types was not a barrier for acceptance. For the first two this aligns with the literature where, although biometric authentication is frequently an appreciated approach, particularly regarding continuous authentication (Dike-Anyiam and Rehmani; [43]; Skalkos et al., [44]; Crawford, et al. [45]), knowledge-based authentication is still sometimes ranked higher (Bolle et al., [46], supporting the PBA/PLA discussion presented earlier. Possession factors (or specifically possession-knowledge hybrids in case of protected smartphone -based approaches), in turn, may have more varied ratings and depend more strongly on the context (Bolle et al., [46]), which indications were supported by the current study. In conjunction with previous research, the current results appear to corroborate the view that for the time being, none of the different types of authentication as such are yet a guarantee of acceptance, and comparisons of individual methods remain important.

#### Individual methos, top cluster: Fingerprint, one-time passcode, and PIN code.

Regarding the individual methods, the high, context-independent popularity of **FP** – despite the concerns voiced in the free answers about its potential readability and security issues – was in line with the high regard seen elsewhere in research in various contexts, for example, in phone unlocking (Cho et al. [33]), mobile banking (Kruzikova et al. [32], website login (Oogami et al. [19]), and card payment (Visa [31]). FP appeared to be well accepted and seen as a highly convenient and relatively safe method. However, when high security was required and financial assets were in question, the ratings of **OTP** were elevated (with frequent references to safety in the free text answers) alongside FP. As such, the regard for OTP in financial context appeared higher than, for example, in the health application context in Arinde et al. [26], where OTP competed for the third place with facial recognition, after both PIN and FP. Overall, the cluster of the three favourites was fairly well in line with the favourites for AoC seen in Arinde et al. [30]. Meanwhile, the popularity of the **PIN** code across contexts in the current study was in line with, for example, the context of mobile banking in Kruzikova et al. [32]. However, a clear dominance of PIN as a favourite, such as seen in the free selection in Arinde et al. [30] in a health system login context, or considerable fluctuations in the regard of PIN in phone unlocking context, as seen in Cho et al. [33], were not observed in the current study. Instead, the current findings, with the tendency of FP to have the highest ratings along with its significant lead for perceived ease of use, support the results in Bhagavatula et al. [20], where FP was found to be preferred over PIN for phone unlocking. Meanwhile, the difference between fingerprint and PIN in Bhagavatula et al. [20] was stronger than in the current study, where PIN was close to or statistically similar to fingerprint, depending on the scenario. Overall, although all three methods were supported for account login and payment confirmation in the current study, the views toward FP appeared to be more cautious in these contexts compared to phone unlocking (Cho et al. [33] Bhagavatula [20]). This could be understandable due to the more continuous need of authentication for smartphone use, weighing more on the requirement for ease of use, which could compromise security – a problem that continuous authentication approaches such as [44] and [45] are attempting to address. Meanwhile, the differences between the three approaches in the login and payment context appear fine on the general level and would invite the division of further research into highly specific scenarios and vignettes.

#### Individual methos, lower cluster: Finger swipe and voice recognition.

**Finger swipe** was clearly less popular than PIN, aligning with the order of frequency of use seen in Cho et al. [33] but with a sense of a lower regard for FS patterns for login and payment than that seen in Cho et al. [33] for phone unlocking. The participants’ reasons for the low popularity of FS appeared more ambiguous than the rationale behind the other methods, with fewer significant associations observed between the considered factors and the evaluations of FS. Experience was not presumed to contribute to the ambiguity since for VR, familiarity and frequency were lower than for FS but the rationales were clearer. Particularly, unlike with the other methods where security was consistently positively linked with favouring the method, for FS this association was only seen for acceptance but not for actual selection of FS. This could suggest that while the participants may have considered FS as a potentially acceptable option in theory, their genuine trust on it may be fragile. This aligns with Cho et al. [33] where, although FS was the third (out of 6 methods in the first, and out of 7 methods in the second study) in the preference rankings, it was the last regarding security in the first study and second last in the second study. The users’ concerns are justified by research such as Andriotis, et al. [47] and Ibrahim et al. [48], which confirmed that while the method benefited from ease of use, it was vulnerable due to weak or predictable patterns selected by the users. This would suggest that for developers applying swipe pattern approaches, security aspects would be particularly important to enhance in the design and, in agreement with [48], user awareness of safe patterns should be enhanced.

**Voice recognition**, although statistically similar to FS, had the tendency of lowest mean rankings, accompanied by participants’ emphasised concerns for reliability/ security as reasons for rejection. The results support both the order and concerns seen in the second study of Cho et al. [33] and the remarks of Renz et al. [34] of low user perceptions of reliability of voice authentication. Meanwhile, in contrast with Renz et al. [34], also perceptions of higher reliability/ security were observed when VR was compared to FS: These were the second most common reason for acceptance of VR. Higher perception of security also correlated significantly with higher likelihood of selecting VR for both login and payment purposes. Meanwhile, a number of assumptions and misconceptions of VR were noted. The findings suggest that for VR systems that have previously been linked with perhaps low perceptions of reliability [31,49], informing audience about the reliability and security of the method may be particularly important, alongside audience-adjusted information about the correct use and operation of the method. Particularly the difference between VR systems requiring recognisable authentication content (such as passwords or fixed phrases) and applications using steganography (using the qualities of voice while hiding the presence of concealed information; e.g., Phipps, et al. [49]) would be important to convey clearly.

#### Perceptions on authentication of choice.

**AoC** was fairly infrequently selected as a preferred method for login or payment when the three favourites were also available as options. In Arinde et al. [30], AoC was contrasted against the use of password and was clearly preferred, even when the use of AoC increased in complexity. However, Arinde et al. [30] did not compare AoC against using single non-PBA methods. Combined, the findings seem to point towards a preference of convenience over several actions or decisions to make, rendering a predefined relatively well accepted method more popular than a choice made by the user in the authentication situation. On the other hand, compared to the cluster of the less popular predefined options in the current study (VR and FS) and to the use of passwords in Arinde et al. [30], AoC was preferred in both cases, suggesting that the users are willing to accept some level of inconvenience in order to use methods perceived as more reliable/ secure or to avoid the use of passwords. Overall, while AoC is a topic of growing research, the findings seem to suggest that for the time being, on average, the users see the choice required in AoC as somewhat inconvenient but also as a way to enhance the security where needed. To further develop and enhance the uptake of AoC options, the ease of use and clarity and simplicity of the choices would therefore be pivotal aspects.

### Justification, trade-offs, and expertise

#### Justification in the numeric data.

Considering the users’ justification of acceptance or rejection overall, across the methods a dominant reason for acceptance was convenience, which supports earlier work, for example Abdulkareem and Gordon [50] regarding biometrics, Grindod, et al., [51] for knowledge-based approaches, and De Cristofaro, et al. [52] considering OTP. Reliability/ security was in many cases not far behind convenience for acceptance, supporting [46]. In contrast, for rejection the most common reason was low reliability/ security, accompanied by device dependency particularly regarding OTP and fingerprint. Further, when the statistical associations between perceived *Ease of use* and *Security* and participants’ choice (*acceptance/ preference*) were investigated, the findings suggested a slightly stronger role for security than for usability.

#### Justification in the free text comments.

The themes emerging in the free-text comments supported the insight from the numeric data, with the comments frequently highlighting concerns around security. The next common aspects were usability issues for OTP, PIN, and FS, and convenience alongside concerns on reliability, accuracy and adaptability, for FP and VR. Meanwhile, the emergence of security issues in the free text answers was in contrast with the findings in Cho et al. [33], where security was not emphasised in the free text responses. Meanwhile, both Cho et al., [33] and the current study observed participants’ comments revealing concerns about accuracy and usability issues across the methods; user memory requirements in knowledge-based methods; risks of fingerprint copying/ misuse during sleep; finger swipe and PIN shoulder surfing; challenges of biometrics between family members (face recognition in Cho et al. [33] and fingerprint in the current study); and social awkwardness regarding biometrics (facial recognition and iris scanner in Cho et al. [33] and voice recognition in the current study). In the current study, the participants also highlighted notions such as storage risk issues with biometrics; key logger risks and lack of complexity and dynamism with PIN; device dependency and requirements with OTP, fingerprint, and finger swipe; and swiping errors with finger swipe patterns.

#### Trade-offs and alignment with technology acceptance models.

While low perceived security and reliability are not frequently cited in the literature as an explicit key reason for *rejecting* an authentication method (with low convenience being a prevalent one (e.g., Hammod and Ali [53], the results emphasise their important role in parallel with convenience.

This supports the discourse in the literature, of a careful balance in trade-offs between convenience, usability, and security (Al-Sarayreh et al. [54], Ambore [55], Furnell et al. [56], Weir et al., [23], DiNocera et al. [57]). Meanwhile, although the results highlight the users’ strong wishes for convenience together with their high regard for security, their views about the current level of security among the methods were occasionally highly mixed – particularly regarding OTP, FP, and VR. It was apparent that the participants’ level of knowledge around the methods can vary extensively, and lacking or misleading information can contribute to their judgement in a crucial way, as seen, for example, in the case of VR. The situation resembles the lower than expected awareness of authentication methods noted by [58] and more recently confirmed by [59] and highlights the importance of effective, user-tailored dissemination of information on the methods.

From a theoretical viewpoint, these key aspects align with the Technology Acceptance Model (TAM) [60] when considering convenience as the Perceived Ease of Use (PEOU) factor, security/reliability as a case of the Perceived Usefulness (PU), and knowledge/information as a key external variable influencing both PEOU and PU, which both influence attitudes towards using the authentication methods. In terms of the further developed Unified Theory of Acceptance and Use of Technology (UTAUT) [38], convenience arguably corresponds with Effort Expectancy (EE), security/reliability with Performance Expectancy (PE), and knowledge/information can be seen as Facilitating Conditions (FC); together these three affect the behavioural intention to adopt the authentication methods. The alignment, in both models, highlights the role of information in influencing the acceptance of authentication methods.

#### Biometric authentication and expertise in the use of information technology.

Considering the clear value of information on the acceptance of these approaches, prior expertise in the field could be expected to play a major role. However, the findings were mixed regarding links between expertise on IT use and views on biometric authentication. Significant although weak associations were found between expertise and regard for the method in case of both FP and VR, but only in part of the scenarios and of opposite types: For FP, the association between expertise and acceptance (login) was positive, while for VR, correlation between expertise and preference (payment) was negative. Mixed results on biometrics have been seen elsewhere; for example, Wolf et al [22], noted a general tendency of experts’ lower trust on biometrics but overall higher readiness to adopt biometric approaches. Nevertheless, the general scarcity of significant associations between expertise and evaluations in the current study would suggest that IT expertise as such is not commonly linked with higher or lower evaluations of authentication methods, whereas for experts specifically on authentication, as suggested in Wolf et al. [22], the case may be different. The importance of providing specific, detailed and user-tailored information is therefore further emphasised among various audiences, including IT experts.

### Recommendations

Careful design and adjustment of information and dissemination about authentication methods to targeted audiences and general public is expected to be a crucial factor in the successful roll-out of PLA systems and is recommended in the promotion and implementation of the systems, to increase their uptake. For biometrics, this applies particularly to **VR**, where misconceptions were present; the promotional materials should, ideally, clearly explain the strengths (e.g., convenience, speed) and the risks/limitations of the method (e.g., vulnerability to spoofing, environmental noise, voice changes); help understand the process and requirements to set up the system; communicate how the data is managed (e.g., storage, encryption); emphasise correct usage, such as using a good quality voice sample, avoiding background noise, ensuring the system captures sufficient voice variation, and following instructions for voice templates; and include audience-adjusted information about the operation of the method. Particularly the difference between VR systems requiring recognisable authentication content (such as passwords or fixed phrases) and applications using steganography (using the qualities of voice while hiding the presence of concealed information) (e.g., [49]) would be important to convey clearly.

Similarly, regarding the other biometric method **FP**, although well perceived, lingering uncertainties and knowledge gaps should be addressed. Promotional materials should explicitly address what fingerprint authentication does and does not guarantee (to correct misconceptions about infallibility); explain common threats (e.g., spoofing, sensor failure, device theft or loss) and limitations (e.g., accuracy between family members; issues with injuries); explain data management and system setup; and in order to manage potentially high expectations, include realistic comparisons to other methods.

While many of the points above (e.g., data management, comparison, setup) also apply for the rest of the methods, in case of the **PIN** code, carefully designed dissemination regarding particularly new, different PIN approaches is expected to be crucially important for their acceptance, due to participants’ wide prior experience of using earlier PIN approaches and the related pre-existing views which were accompanied by various concerns (e.g., simplicity, static approach, shoulder surfing and key loggers, memory requirements). To address these, the differences between the new and the familiar approaches would require clear, user-centred explanations. Meanwhile, in case of traditional PINs, particular attention should be paid to emphasise safe usage (e.g., avoiding predictable patterns, not reusing/ sharing/ accidentally showing the PIN, clearing traces of the PIN on the device, and updating the PIN and where applicable, considering several layers of protection instead of enabling access to multiple sensitive applications through one PIN code).

For both **OTP and FS**, device dependency, and in case of OTP, also network dependency, emerged as concerns important to address in their development and dissemination. In addition, for OTP, other aspects to address contained, e.g., delays, mix-ups with resent OTPs, annoyance when needed repeatedly, and situations of awkward transfer from the reception point to the target solution. Meanwhile, the benefits seen by the users of, e.g., low memory requirements, quick expiration, and information value (if someone else is attempting access) should be emphasised.

Particular aspects to address regarding FS would include predictability of too simple patterns contrasted with the memory requirements of more challenging ones, shoulder surfing, mistakes in swiping, broken screen, and, considering different implementations, background-guided (e.g., dots) option’s predictability vs challenges of a potential free-form approach. Moreover, clear explanation of the operation and use of FS would be important due to some misconceptions.

Finally, to further develop and enhance the uptake of AoC approaches, the ease of use at the point of choice and the clarity and simplicity of the available options to choose from would be pivotal aspects to address both in design and in promotional materials, to reduce the concern of cognitive burden at the point of authentication.

Finally, the issues and concerns raised in the free-text comments overall are recommended to be addressed more widely, where applicable, in the development and in the roll-out information campaigns of new PLA approaches of any type. Particularly, it should be ensured that the audiences are sufficiently aware of the ways in which security issues have been resolved or improved between the old and the new approaches.

### Limitations and future research

Although the order of presentation of the methods in the first part of the survey was predefined, this was not deemed likely to have changed the conclusions, since the random-ordered.

evaluations in the second part of the survey followed a very similar pattern than the evaluations in the first part, and this pattern was different from the order of presentation. In addition, evaluation of each method was independent of the others, and participants were able to return to review and edit their answers at any point. The order of presentation was functional for the evaluation process: It placed a well-known method first to help participants engage with the survey, while it separated similar methods from each other, to avoid confusion. In addition, very few answers were obligatory, and although this may have led to some missed data, it aimed to reduce potential effects of frustration or tiredness at the point of later methods in this section. Nonetheless, to minimise any potential impact of presentation order, further research might use random ordering also in the first section. Future research might also include more use cases such as face recognition, key fob, push notification, and email magic link, and involve further authentication scenarios, to increase comparability. The inclusion of further aspects, however, requires balancing the number of questions and the use cases to evaluate, which were both seen as relatively high for the participant engagement already in the current survey. For this reason, for example, the lists of pre-stated reasons for acceptance or rejection were limited. However, the option of expressing further rationale through free text was included at each point to ensure consideration of any further views. In addition, to facilitate and speed up the completion of the survey and therefore enhance the likelihood of participants’ completion of the method evaluation questions, the two more complex potential reasons for acceptance or rejection in the first part of the survey, reliability and security, were addressed together under one combined option. Meanwhile, the differentiation between these reasons was possible through the analysis of free-text comments, where they emerged separately, and through the evaluation instrument addressing perceived security alone, which both supported a strong role of the security aspects. Nevertheless, future research could aim to explore these concepts separately also in multiple choice rationale sections, as long as the concepts are clearly defined to the participants; however, this is expected to increase the time requirements of the survey. Further related to sufficient clarity of the questions, possible simplification of some of the wording of the questions could be investigated and tested, and the presentation of the methods could be improved by using short video clips of the authentication situations, to ensure optimally equal understanding of the use cases and address the rare cases of apparent misconceptions amongst the participants. Although revealing and important, in the current study these cases were few compared to the large majority that indicated understanding in line with the method presentations and are therefore not estimated to have changed the conclusions. Meanwhile, in case of video clips, accessibility issues would need consideration. Regarding generalisability and accuracy, research with larger data sets would be able to narrow down the margins of error in the current study, where comparisons across the participants covered a sample size of 129–142 participants, depending on the aspect. Although the sample size exceeded the minimum target and a higher than minimum confidence level (95%) could be applied, the margins of error did occasionally reach the upper limit of the target range, and further accuracy from larger data sets would be valuable particularly for exploring the finer differences between OTP, FP, and PIN, or, on the other hand, VR and FS, in the future. Finally, it was noted that evidence in the literature remain scarce on a topic related to the dissemination of authentication approaches: More data would be needed on how exactly consumer-oriented material should present the risks of different authentication methods, for the best outcomes and increased uptake of the approaches. Overall, future research could explore in detail the types and levels of information that different kind of users find most useful particularly on the key topics of security and convenience, considering different approaches to authentication.

### Contributions

The findings of the current study respond to a gap in knowledge regarding the integration of views of end-users in the design and roll-out of end-user authentication systems [16,18] and enhances the coverage of data on users’ views on the wide range of possible authentication methods and contexts. Previous studies have explored a range of PLA methods separately – for example, the increasingly used fingerprint [19,31] or promising FIDO2 solutions [16] – and in varying combinations [32–34], in separate contexts such as phone rebooting and unlocking (20, 33, credit card payments [31], website login [19], and mobile banking [32]. Meanwhile, the current study extended the investigation of user views to the comparison of one-time passcode, fingerprint, voice recognition, personal identification number, finger swipe, and authentication of choice, and to the contexts of low-risk account login and higher risk payment confirmation, observed in parallel.

The results can guide the design of user-centred and therefore optimally higher-uptake PLA approaches, informed by the users’ perceptions – including misconceptions – of the concerns and benefits of OTP, FP, VR, PIN, FS, and of selecting a method at the point of authentication, in different contexts. They can guide the design of the information campaigns related to promotion and roll-out of authentication methods, providing insight on existing preconceptions to address and on important aspects to emphasise. They can help consumers in the public, businesses and third sector organisations, to consider and select a suitable option between different authentication approaches. They can also help researchers better understand the rationales behind users’ behaviour regarding the approaches investigated, including risky practices linked to vulnerabilities of authentication methods; in addition, they provide insight on the perceptions of users regarding other users’ behaviour.

The findings are valuable in the current situation where, as Haddad et al. [13] posit, the apparently secure nature of PLA use cases as compared to PBA methods has attracted the support of, and deployment by, larger organisations such as Microsoft and Google, while several of the users’ concerns on PLA [16] remain to be solved.

The findings highlight the importance of responsibly innovative PLA systems and design, involving a culture of research and development that integrates the evolving perceptions of end-users into system design and roll-out, leading to a wider adoption and utilisation of PLA approaches in digital systems.

## Conclusion

The current study evaluated the views of end users on five different authentication methods and authentication of choice, and across two types of authentication situations, observing also the users’ experience on IT. A cluster of favourites was formed by OTP, FP, and PIN, which were commonly statistically similar, although fingerprint had occasional advantage over the others. AoC was placed between this top cluster and the second group formed by VR and FS, which were also statistically similar to each other. Among the reasons behind the participants’ evaluations, dominant factors were convenience and usability from a positive viewpoint, and security issues, reliability and accuracy as concerns, with a range of specific issues expressed in the free-text answers. Knowledge gaps and misconceptions were present, highlighting the importance of carefully designed, adjusted, and targeted information to be linked with any authentication system roll-out.

The results of this study are expected to benefit the industry involved in identity and access management, serving both individuals and corporate entities, by providing empirical evidence on the views of the users on the addressed authentication systems. Taking these outcomes into consideration is suggested for the relevant industries and stakeholders when developing end-user authentication systems, especially PLAs; this is expected to lead to a wider adoption of these solutions in computer-based user authentication contexts. Finally, the findings are expected to enhance the knowledge of professionals and industry experts specialising in user experience design and management, in terms of the current and developing perceptions of users on authentication methods and their related use issues.

## Supporting information

S1 Appendix Evaluation questions. (DOCX)

S2 Appendix Expertise on IT use. (DOCX)

S3 Appendix Method presentations. (DOCX)
