# Supplementary material for: Comparing perceptions of users on digital authentication through one-time passcode, fingerprint, voice recognition, PIN code, finger swipe, and authentication of choice: A cross-sectional survey
Source: PLoS One. 2026 Apr 1;21(4):e0344162. doi: 10.1371/journal.pone.0344162 (PMC13042729; doi:10.1371/journal.pone.0344162)
Supplement: S1 Appendix — (DOCX) [file pone.0344162.s001.docx]

## Evaluation questions

After presentation, each authentication method was evaluated using the following questions:

- *How familiar are you with this type of authentication? (Response scale: Not familiar at all - Extremely familiar (5-point scale))*
- *Have you used this type of authentication before? (Response scale: Never - 5 to 7 days a week (5-point scale)/Not sure)*
- *Are you/would you be happy using this method of authentication, instead of a password, for logging in to a customer account, e.g., to an online shop? (Response scale: No, very unhappy - Yes, very happy (7-point scale))*
- *Are you/would you be happy using this method of authentication to confirm payments from your account, e.g., while banking or shopping online? (Response scale: No, very unhappy - Yes, very happy (7-point scale))*

The rationale for the willingness to use the method was asked using the following questions:

- *Please tell more about why you would not be happy using this method, instead of a password for logging in to a customer account, e.g., to an online shop/to confirm payments from your account, e.g., while banking or shopping online] (choose as many as you wish)?*
- *Too slow*
- *Too much required to set it up.*
- *Seems too unreliable/insecure.*
- *Too complicated to use.*
- *Too unfamiliar*
- *Too dependent on the device needed for authorisation.*
- *Not convenient to use.*
- *I'm not sure.*
- *Other, please state.*
- *Please tell more about why you would be happy using this method, instead of a password for logging in to a customer account, e.g., to an online shop / to confirm payments from your account, e.g., while banking or shopping online] (choose as many as you wish)?*
- *Fast*
- *Convenient*
- *Seems reliable/secure enough.*
- *Familiar*
- *Easy to use.*
- *Not too dependent on the device*
- *I'm not sure.*
- *Other, please state.*

Any participants who expressed that they would be somewhat to very unhappy to use the method in both the login and the payment confirmation situation, were asked whether they thought there was anything positive about the method:

- *Do you see anything positive or beneficial about this method of authentication (choose as many as you wish)?*
- *Fast*
- *Convenient*
- *Seems reliable.*
- *Familiar*
- *Easy to use.*
- *Not too dependent on the device*
- *I'm not sure.*
- *Not really*
- *Other, please state.*

Respectively, any participants who had been somewhat to be very happy to use to use the method in both the login and the payment confirmation situation were asked whether they thought there was anything negative about the method:

- *Do you see anything negative or problematic about this method of authentication (choose as many as you wish)?*
- *Too slow*
- *Too much required to set it up.*
- *Seems too unreliable/insecure.*
- *Too complicated to use.*
- *Too unfamiliar*
- *Too dependent on the device needed for authorisation.*
- *Not convenient to use.*
- *I'm not sure.*
- *Not really*
- *Other, please state.*

The participants were offered the opportunity to add further comments about the method through an open-ended question:

- *Is there anything further you would like to add about this method?*

The participants were asked to rank the methods of authentication and give their preferences, with the options presented in random order:

- *Please rank these methods of authentication in your order of preference, considering how easy you feel they are to use (Write '1' for first choice, '2' for second, etc.)*
- *Please rank these methods of authentication in your order of preference, considering how secure you feel they are (Write '1' for first choice, '2' for second, etc.)*
- *What method would you most like to use, instead of a password, for logging in to a system as a customer, e.g., when logging into an online shop? (Choose as many as you wish)*
- *What method would you most like to use for confirming payments online, e.g., while banking or shopping? (Choose as many as you wish)*

Finally, the participants were asked their gender, age, and their use of information technology:

- *Which of the following best describes your use of information technology (IT)?*
- *Occasional IT user (e.g., irregular Internet use, email, browsing)*
- *Frequent IT user (e.g., frequent Internet use, communication, photos, games, or other applications)*
- *Using IT in everyday work or projects, for standard processes (e.g., writing, spreadsheets, meetings, image editing, etc.)*
- *Using IT in everyday work or projects, for complex processes (e.g., basic data collection & analysis, databases, design, guided programming etc., but not for development of IT-related processes or tools)*
- *Everyday work or projects on an IT developer level / IT professional (e.g., advanced programming or analysis / advanced process or tool development / advanced problem solving / IT software infrastructure, etc.)*
- *Prefer not to say.*

The survey ended with an open question about any further comments:

- *Thank you for participating. Is there anything you would like to add or comment?*
