# Supplementary material for: Comparing perceptions of users on digital authentication through one-time passcode, fingerprint, voice recognition, PIN code, finger swipe, and authentication of choice: A cross-sectional survey
Source: PLoS One. 2026 Apr 1;21(4):e0344162. doi: 10.1371/journal.pone.0344162 (PMC13042729; doi:10.1371/journal.pone.0344162)
Supplement: S2 Appendix — (DOCX) [file pone.0344162.s002.docx]

## Expertise on IT use

Encoding of the use of IT, five levels:

Level 1 Occasional IT user (irregular Internet use, email, browsing)

Level 2 Frequent IT user (frequent Internet use, communication, photos, games, consumer applications)

Level 3 Using IT in everyday work or projects, for standard processes (writing, spreadsheets, meetings, data editing

Level 4 Using IT in everyday work or projects, for complex processes (basic data collection & analysis, databases, design, guided programming, but not development of IT-related processes or tools)

Level 5 Everyday work or projects on an IT developer level / IT professional (advanced programming or analysis / advanced process or tool development / advanced problem solving / IT software infrastructure)
