# Supplementary figures and images for: Comparing perceptions of users on digital authentication through one-time passcode, fingerprint, voice recognition, PIN code, finger swipe, and authentication of choice: A cross-sectional survey
Source: PLoS One. 2026 Apr 1;21(4):e0344162. doi: 10.1371/journal.pone.0344162 (PMC13042729; doi:10.1371/journal.pone.0344162)

## Method presentations


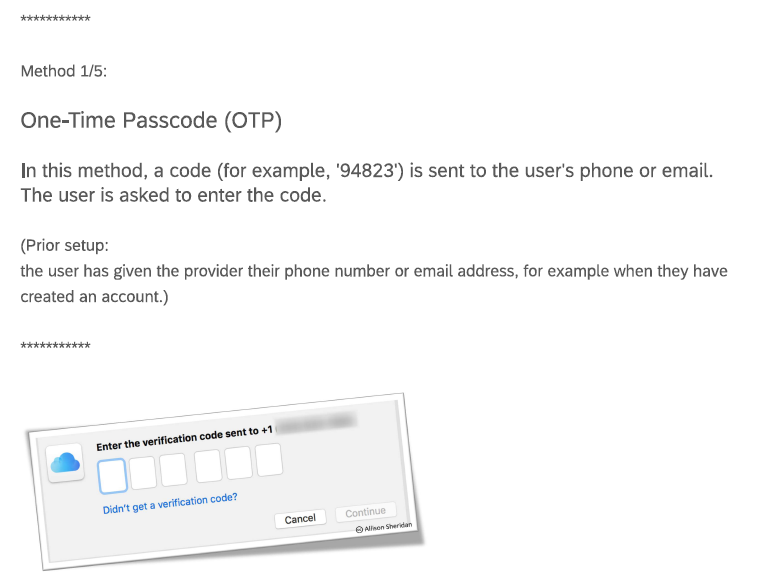


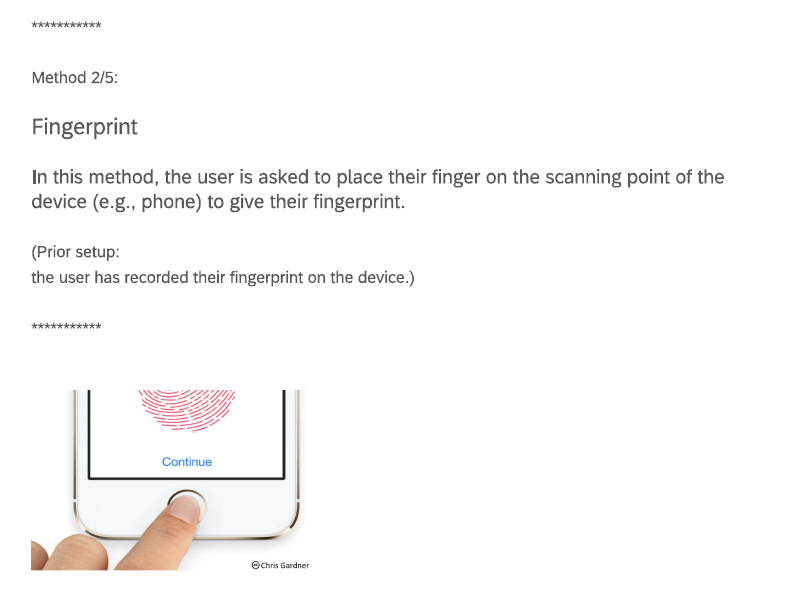


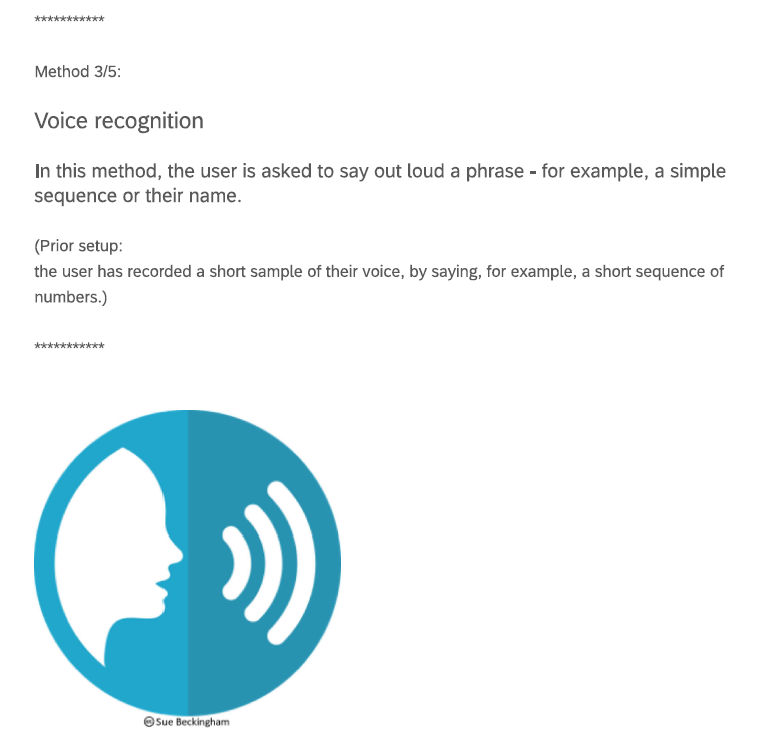


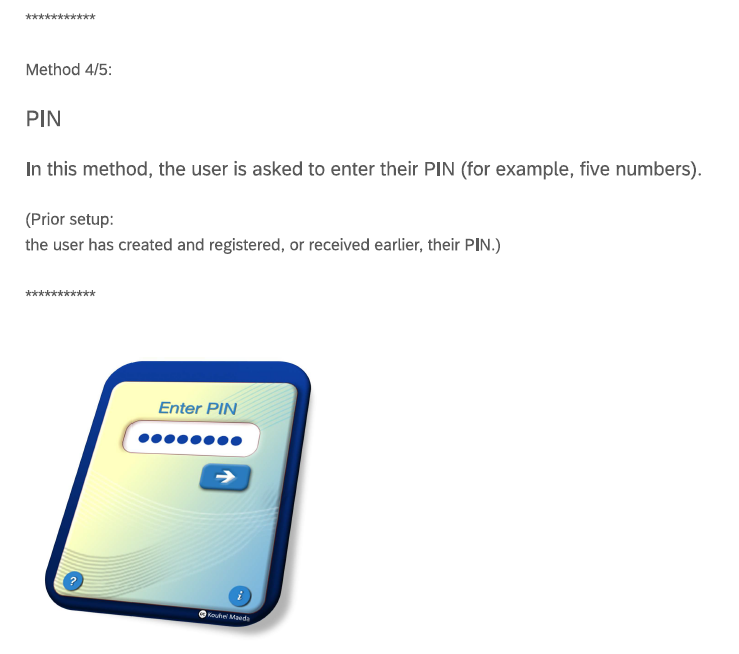


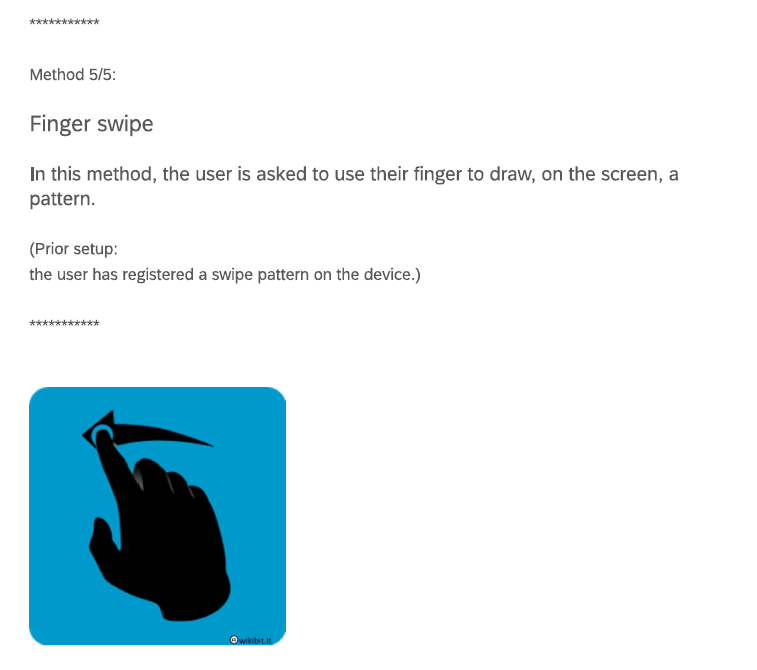

Supplement: S3 Appendix — (DOCX) [file pone.0344162.s003.docx]
